# A conserved ATG2‐GABARAP family interaction is critical for phagophore formation

**DOI:** 10.15252/embr.201948412

**Published:** 2020-02-03

**Authors:** Mihaela Bozic, Luuk van den Bekerom, Beth A Milne, Nicola Goodman, Lisa Roberston, Alan R Prescott, Thomas J Macartney, Nina Dawe, David G McEwan

**Affiliations:** ^1^ Division of Cell Signalling & Immunology School of Life Sciences University of Dundee Dundee UK; ^2^ Edinburgh Cancer Research UK Centre MRC Institute of Genetics and Molecular Medicine University of Edinburgh Edinburgh UK; ^3^ MRC Protein Phosphorylation and Ubiquitylation Unit School of Life Sciences University of Dundee Dundee UK; ^4^ Dundee Imaging Facility School of Life Sciences University of Dundee Dundee UK; ^5^ Cancer Research UK Beatson Institute Glasgow UK

**Keywords:** ATG2, autophagosome, autophagy, GABARAP, phagophore, Autophagy & Cell Death

## Abstract

The intracellular trafficking pathway, macroautophagy, is a recycling and disposal service that can be upregulated during periods of stress to maintain cellular homeostasis. An essential phase is the elongation and closure of the phagophore to seal and isolate unwanted cargo prior to lysosomal degradation. Human ATG2A and ATG2B proteins, through their interaction with WIPI proteins, are thought to be key players during phagophore elongation and closure, but little mechanistic detail is known about their function. We have identified a highly conserved motif driving the interaction between human ATG2 and GABARAP proteins that is in close proximity to the ATG2‐WIPI4 interaction site. We show that the ATG2A‐GABARAP interaction mutants are unable to form and close phagophores resulting in blocked autophagy, similar to ATG2A/ATG2B double‐knockout cells. In contrast, the ATG2A‐WIPI4 interaction mutant fully restored phagophore formation and autophagy flux, similar to wild‐type ATG2A. Taken together, we provide new mechanistic insights into the requirements for ATG2 function at the phagophore and suggest that an ATG2‐GABARAP/GABARAP‐L1 interaction is essential for phagophore formation, whereas ATG2‐WIPI4 interaction is dispensable.

## Graphical abstract

A direct interaction between ATG2A and GABARAP family of proteins is essential for phagophore maturation. Loss of this interaction results in the accumulation of open and immature vesicles and complete blockage of autophagy flux.

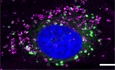

## Introduction

The ability of our cells to deal with a wide variety of cellular stresses depends on two quality control pathways—the ubiquitin–proteasome and the autophagosome‐to‐lysosome (macroautophagy) pathways. Both act in concert to ensure that homeostasis is maintained in our cells. Macroautophagy (henceforth autophagy) is a multi‐step process that requires the initiation and formation of a phagophore that grows and surrounds cargo to be degraded. The phagophore eventually seals to form a double‐membraned vesicle, termed autophagosome. The autophagosome is then transported to, and fuses with, the lysosome where the inner autophagosomal membrane along with the cargo contents is degraded and recycled back to the cell (reviewed in 1). This provides an intracellular pool of amino acids and lipids that the cell can utilize under periods of stress. Autophagy is induced by stresses including amino acid/growth factor starvation (non‐selective, bulk autophagy), mitochondrial depolarization 2, 3, pathogen invasion 4 and protein aggregate accumulation 5 (selective autophagy). In all cases, the inclusion of the cargo within the growing phagophore, and eventually the autophagosome, serves to isolate potentially cytotoxic material from the surrounding intracellular environment.

The molecular machinery involved in autophagosome formation is extensive and, for the most part, highly conserved. More than 30 ATG (autophagy‐related) proteins regulate all stages of autophagosome formation; from initiation, cargo selection, transport and fusion with the lysosome. In higher eukaryotes, several kinase complexes, as well as ubiquitin‐like conjugation machinery, are required for the initiation and expansion of the autophagosome. For example, the initiation kinase complex consists of ULK1/ATG13/ATG101/FIP200 and the lipid kinase complex VPS34/Beclin1/ATG14L1/p150 6, 7, 8. Growth of the autophagosome and cargo recruitment requires the ubiquitin‐like conjugation machinery, consisting of ATG7 (E1‐like) ATG3 and ATG10 (E2‐like) and ATG12‐ATG5‐ATG16L1 (E3‐like complex), which are responsible for the conjugation of ubiquitin‐like MAP1LC3 (microtubule‐associated protein 1A/1B light chain)/GABARAPs (gamma‐aminobutyric acid receptor‐associated proteins; mammalian homologues of yeast Atg8) to phosphatidylethanolamine (PE) on the growing phagophore membrane 9. LC3/GABARAP proteins, once conjugated to PE, can localize to both the inner and outer autophagosomal membranes. This allows the ATG8s to interact with proteins containing an LC3 interaction region (LIR), linking the phagophore to the cargo or the phagophore/autophagosome to the cellular transport and fusion machinery 10, 11, 12, 13. The majority of LIR motifs contain a core W/Y/F‐x‐x‐L/I/V motif. In addition, acidic and/or phosphorylatable serine/threonine residues N‐ and C‐terminal of the core LIR sequence can contribute to the stabilization of LIR‐ATG8 interactions 13, 14, 15, 16.

Despite a surge in our understanding of the mechanisms involved in autophagy, there are still questions pertaining as to how the double‐membrane phagophore closes and seals to form the autophagosome. In particular, the molecular components and how they interact are relatively unknown. For example, in yeast, Vps21 (Rab5‐related GTPase) and Rab5 influence phagophore closure 17, 18. The mammalian ATG8 protein GATE‐16 (GABARAP‐L2) has been shown to be involved in the later stages of autophagosome biogenesis 19 and its N‐terminal extension can promote membrane fusion events, hinting at a possible role during phagophore closure 20. However, a recent study where LC3 and GABARAPs were knocked out indicated that LC3/GABARAPs were not required for phagophore closure 21. A mutant form of ATG4B (C74A), the cysteine protease responsible for LC3 and GABARAP priming and removal from the autophagosomal membrane, prevents LC3 and GABARAP lipidation and results in an increased number of unsealed phagophore membranes 22. In addition to core autophagy proteins, a component of the ESCRT‐III (endosomal sorting complex required for transport) endocytic machinery, CHMP2A, regulates the separation of inner and outer phagophore membranes 23.

One intriguing example of the role of ATG proteins during phagophore formation and closure is the poorly understood ATG2 proteins, ATG2A and ATG2B. Mammalian ATG2s are > 1,900 amino acids in length and share approximately 40% amino acid sequence homology but are only 13% similar to the single isoform of *S. cerevisiae* Atg2 and 24–26% to the *D. melanogaster* Atg2, indicating a potential divergence of function. Indeed, the reconstitution of human ATG2A in yeast Δ*atg2* cells is not sufficient to restore the autophagy defects 24. In yeast, Atg2 constitutively interacts with Atg18 at phosphatidylinositol‐3‐phosphate (PtIns3P)‐rich membrane regions and tethers pre‐autophagosomal membranes to the endoplasmic reticulum for autophagosome formation 25, 26. Mammalian homologues of yeast Atg18 are the WIPI (WD repeat domain phosphoinositide‐interacting) proteins (WIPI1‐4) that are involved in various stages of autophagosome formation 27, 28, 29. ATG2A and ATG2B preferentially interact with WIPI4 (WDR45) through a conserved Y/HFS motif 29, 30, 31. Simultaneous depletion of both ATG2A and ATG2B results in the accumulation of small, open immature phagophore structures 32, 33. The depletion of WIPI4 also causes open phagophore structures, but they are morphologically dissimilar to those generated after ATG2A/B depletion 29. Interestingly, previous studies have not, despite mapping the ATG2‐WIPI4 interaction, shown whether this interaction is required for the restoration of autophagy flux in ATG2A/B‐depleted cells 29, 30, 31. Herein, CRISPR/Cas9 was used to generate GFP‐ATG2A knock‐in cells as a tool to address the endogenous localization and interaction of human ATG2A. We have identified a direct interaction between the GABARAP family of mammalian ATG8 proteins and ATG2A and ATG2B that is mediated through a highly conserved LIR sequence. Surprisingly, the newly identified LIR sequence in ATG2A and ATG2B is approximately 30‐amino acid N‐terminal of the WIPI4 interaction motif and represents independent interaction sites in the C‐terminus of human ATG2s. Using reconstituted ATG2A/2B double‐knockout cells, we show that the disruption of ATG2A‐WIPI4 interaction had no discernible effects on phagophore closure and autophagy flux but slightly enhanced lipidated GABARAP interaction, whereas mutation of the LIR motif on ATG2 completely blocked phagophore closure and autophagy flux, despite ATG2A maintaining its ability to interact with WIPI4. Taken together, these data provide new insights into essential ATG2 interactions during autophagosome biogenesis.

## Results and Discussion

### Endogenous GFP‐tagged ATG2A co‐localizes and co‐precipitates with GABARAP/GABARAP‐L1

**Figure EV1:**
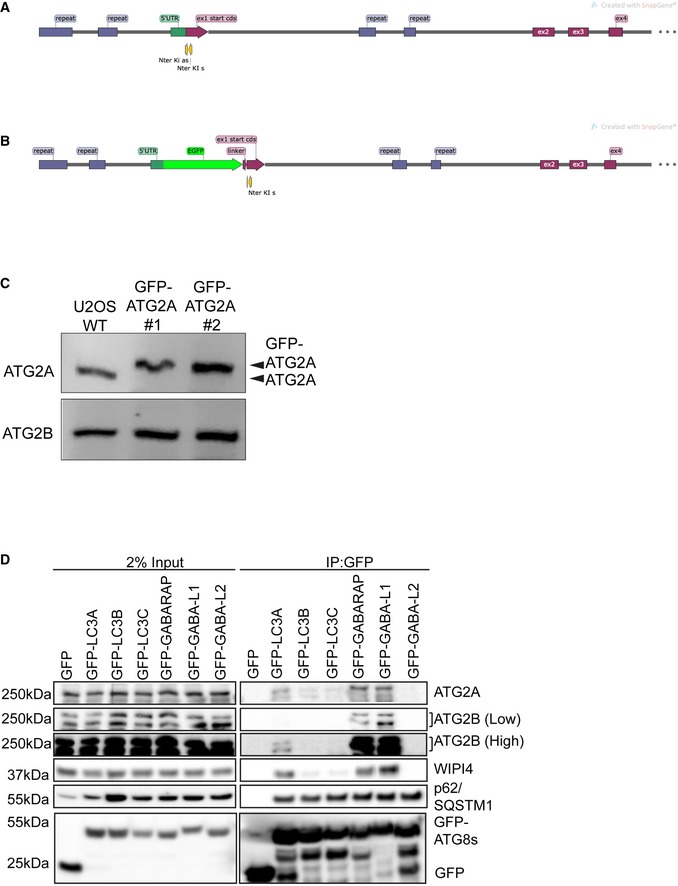
Generation of GFP‐tagged endogenous ATG2A A, BStrategy for insertion of GFP‐tag upstream of human ATG2A exon 1. Graphic shows position of guides and locus before and after (B) GFP‐tag plus linker insertion.CWestern blot of total cell lysates from parental wild type (WT) and GFP‐ATG2A CRISPR/Cas9 knock‐in clones 1 and 2 using anti‐ATG2A and anti‐ATG2B antibodies.DGFP alone or GFP‐tagged mammalian ATG8 proteins (LC3A, LC3B, LC3C, GABARAP, GABARAP‐L1 and GABARAP‐L2) were overexpressed in HEK293T cells, lysed and the GFP‐tag immunoprecipitated using GFP‐TRAP beads. Samples were then run on 4–12% Bis–Tris gel and transferred to PVDF membrane and blotted for the presence of ATG2A, ATG2B, p62/SQSTM1, WIPI4 and anti‐GFP. Blots are representative of *n* = 3 independent experiments. Source data are available online for this figure.

**Figure 1:**
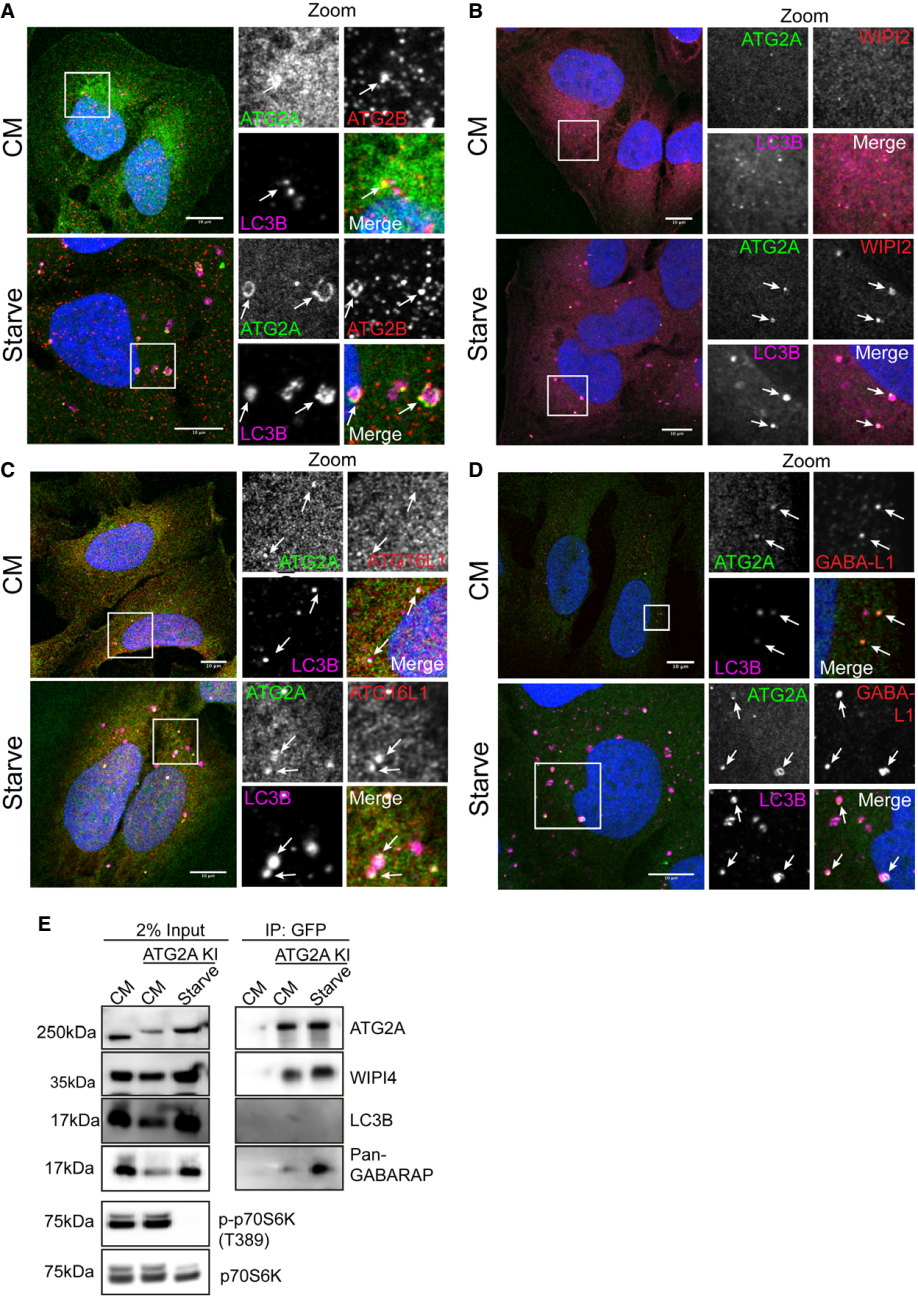
CRISPR/Cas9 GFP‐tagged ATG2A localizes to early autophagy membranes AU2OS cells modified to expressed endogenous GFP‐tagged ATG2A (green) were grown in complete media (CM) or starved (EBSS) for 2 h before fixation and immunostaining with antibodies against ATG2B (red) and LC3B (magenta) and analysed by confocal microscopy. Arrows mark ATG2A/LC3B/ATG2B‐positive structures. Scale bar 10 μm.B–DCells were treated as in (A) and stained with anti‐WIPI2 (red) or (C) anti‐ATG16L1 (red) or (D) anti‐GABARAP‐L1 (red). Arrows mark structures of interest. Scale bar 10 μm.EU2OS WT or U2OS GFP‐ATG2A knock‐in (KI) cells were grown in CM or starvation media for 2 h, lysed and incubated with anti‐GFP nanobody beads coupled to agarose to immunoprecipitate (IP) GFP‐ATG2A. IP samples and 2% input lysates were run on 4–12% gradient gel and processed for Western blotting. Anti‐ATG2A, anti‐WIPI4 and anti‐LC3B, and anti‐GABARAP (pan) were used to probe for the presence/absence of autophagy proteins in the immunoprecipitated samples. p‐p70S6K (T389) was used as a marker for starved cells and total p70S6K as loading control.

In order to study the function of endogenous ATG2 proteins, we generated GFP‐tagged ATG2A knock‐in U2OS cells using CRISPR/Cas9 (Figs EV1A and 1C). Under complete, nutrient‐rich conditions (CM), GFP‐ATG2A showed a dispersed localization, with little overlap with LC3B (Fig 1A, Upper panels). However, upon starvation we observed the formation of punctate and ring‐like structures that localized in close proximity to LC3B‐positive vesicles (Fig 1A, Lower panels). Endogenous ATG2B co‐localized with GFP‐ATG2A on both the punctate and ring‐like structures observed (Fig 1A, lower panels). Furthermore, endogenous GFP‐ATG2A co‐localized with early autophagy marker proteins WIPI2 (Fig 1B) and ATG16L1 (Fig 1C) at LC3B‐positive structures formed under starvation conditions. In addition, GABARAP‐L1 was present on GFP‐ATG2A/LC3B‐positive structures under starvation conditions (Fig 1D). Given the presence of both GABARAP‐L1 and LC3B co‐localizing with GFP‐ATG2A, we were curious as to whether we could co‐precipitate an endogenous ATG2A‐LC3/GABARAP complex using GFP‐ATG2A as bait. Using U2OS WT (control) or GFP‐ATG2A U2OS cells under CM or starvation conditions, we immunoprecipitated GFP‐ATG2A. WIPI4, a cognate ATG2 interaction partner 29, 30, 31, co‐precipitated with GFP‐ATG2A under both CM and starvation conditions (Fig 1E). We could not detect endogenous LC3B in GFP‐ATG2A immunoprecipitates, but we detected increased co‐precipitation of GABARAP proteins, using a pan‐GABARAP antibody under starvation conditions (Fig 1E). Endogenous ATG2A and ATG2B were able to co‐precipitate with GFP‐tagged GABARAP, GABARAP‐L1 and weakly with LC3A but not with GFP‐LC3B, GFP‐LC3C or GABARAP‐L2 when overexpressed in HEK293T cells (Fig EV1D). Notably, endogenous WIPI4 co‐precipitated with GFP‐ATG8s only when ATG2A or ATG2B proteins were present, indicating a potential complex between ATG2, WIPI4 and the ATG8s. Given that we could detect endogenous GABARAP proteins co‐localizing and co‐precipitating with ATG2s, we hypothesize that these form the functionally active complex. However, we cannot rule out a role for LC3A, but we have been unable to confirm an endogenous complex between ATG2, LC3A and WIPI4 proteins.

### Identification of a conserved LC3 interaction region in ATG2A and ATG2B

**Figure EV2:**
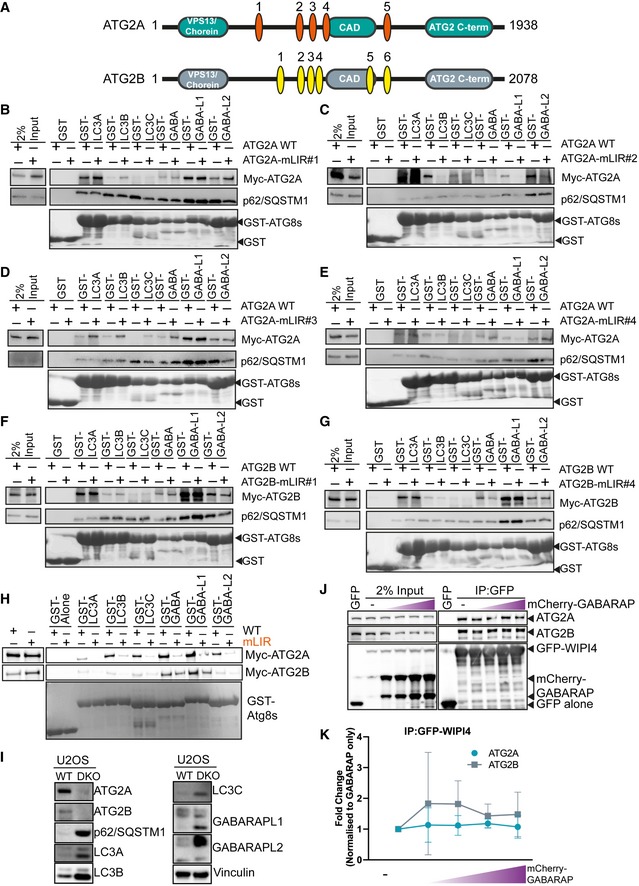
ATG2A and ATG2B contain a highly conserved LC3 interaction region (LIR). Related to Fig 2 ADomain structure of ATG2A (green) and ATG2B (grey) proteins. Both ATG2s contain an N‐terminal VPS13/chorein domain, ATG2 C‐terminal autophagy domain (CAD motif) and ATG2 C‐terminal domain. Position and sequence of putative ATG2 LC3 interaction regions (LIRs) as identified by iLIR and manual annotation. ATG2A has 5 potential LIRs, and ATG2B has 6 potential LIRs. See Table 1 for details.B–EMyc‐tagged ATG2A wild type (WT) and putative LIR mutants, where the potential core motif was mutated to alanine, were used in a pull‐down assay with GST‐tagged mammalian ATG8 proteins. Shown are ATG2‐mLIR#1 (B), ATG2‐mLIR#2 (C), ATG2‐mLIR#3 (D) and ATG2‐mLIR#4 (E). ATG2‐mLIR#5 is shown on (H). ATG2A‐WT or ATG2A‐mLIRs were overexpressed in HEK293T cells, and lysates were incubated with purified GST alone or GST‐tagged LC3A, LC3B, LC3C, GABARAP, GABARAP‐L1 or GABARAP‐L2. Samples were spun, washed and blotted for the presence/absence of Myc‐tagged ATG2A using anti‐Myc antibody. Anti‐p62/SQSTM1 was used as an internal control for the GST pull‐down samples. GST proteins were visualized by Ponceau S staining of membranes.F, GAs in (B) but using Myc‐tagged ATG2B‐WT or ATG2B‐mLIR proteins. Shown are ATG2B mLIR#1 (F) and ATG2B mLIR#4 (G). ATG2B‐mLIR#2 was present on an alpha helix, whereas ATG2B‐mLIR#3 and ATG2B‐mLIR#5 were not expressed. Anti‐p62/SQSTM1 was used as an internal control for the GST pull‐down samples. All blots are representative of at least *n* = 3 independent experiments.HMyc‐tagged ATG2A wild type (WT), ATG2A‐mutant LIR #5 (mLIR; FCIL/AAAA) (upper blots) or Myc‐tagged ATG2B‐WT or ATG2B‐mLIR (FCIL/AAAA; lower blots) were overexpressed in HEK293T cells, and lysates were incubated with purified GST alone or GST‐tagged LC3A, LC3B, LC3C, GABARAP, GABARAP‐L1 or GABARAP‐L2. Samples were spun, washed and blotted for the presence/absence of Myc‐tagged ATG2s using anti‐Myc antibody. GST proteins were visualized by Ponceau S staining of membranes.IU2OS WT and ATG2A/B double‐knockout cell total lysates, analysed for the presence/absence of autophagy marker proteins including ATG2A, ATG2B, p62/SQSTM1, LC3A, LC3B, LC3C, GABARAP‐L1, GABARAP‐L2 and vinculin used as loading control.JGFP alone, GFP‐WIPI4 or GFP‐WIPI4 with increasing concentrations of mCherry‐GABARAP was expressed in HEK293T cells for 24 h, lysed and GFP‐TRAP beads used to immunoprecipitate GFP alone or GFP‐WIPI4. Samples were then probed with antibodies to detect endogenous ATG2A or ATG2B, anti‐GFP and anti‐GABARAP. Blots are representative of *n* = 3 independent experiments.KQuantification of ATG2A (blue line, round symbols) and ATG2B (grey line and squares) co‐precipitation with GFP‐WIPI4 in the presence of increasing concentrations of mCherry‐GABARAP from (J). Co‐precipitation was normalized to GFP‐WIPI4 alone. Line and error bars are mean ± SD of *n* = 3 independent experiments.

**Figure 2:**
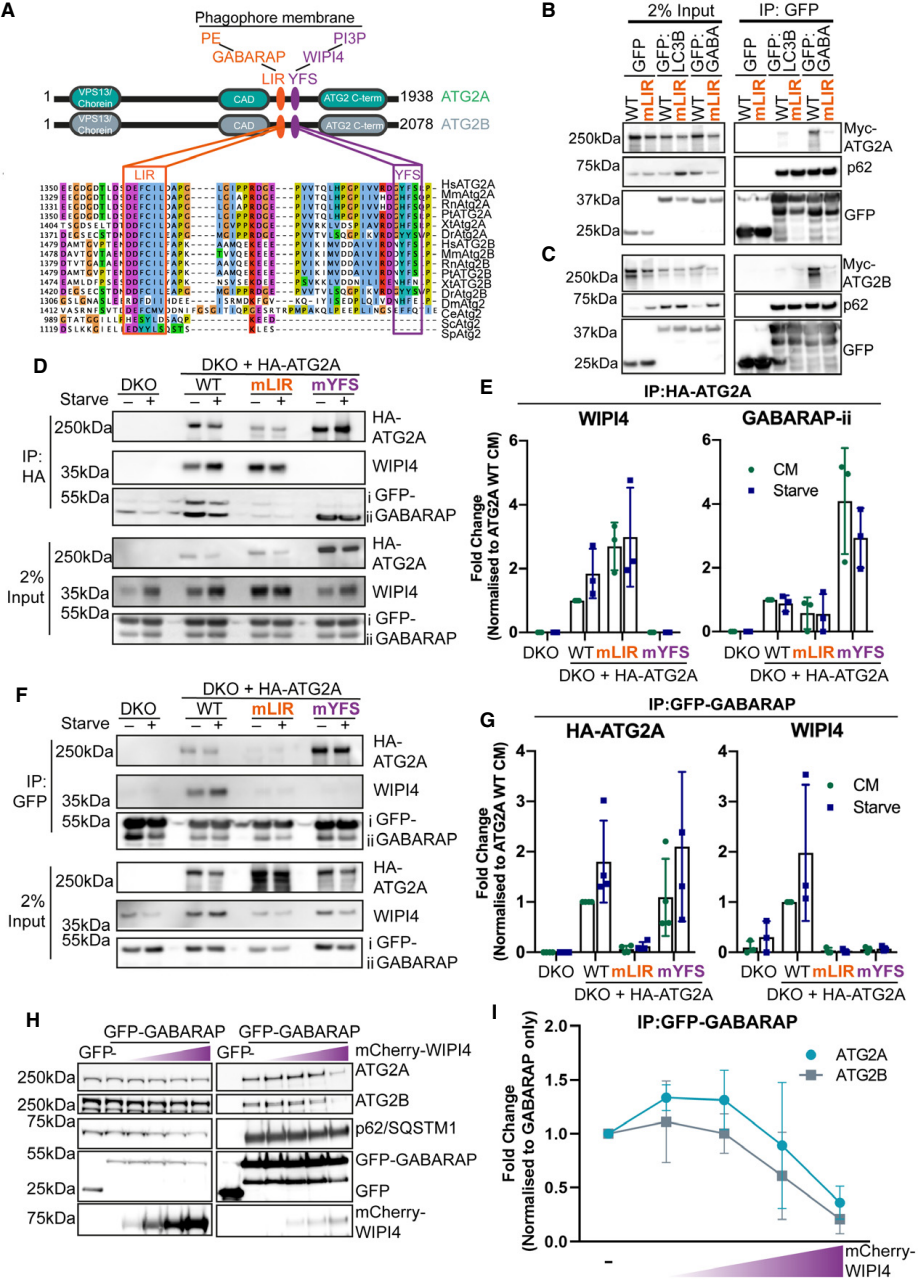
ATG2A and ATG2B contain a highly conserved LC3 interaction region (LIR) Domain structure of ATG2A (green) and ATG2B (grey) proteins. Both ATG2s contain an N‐terminal VPS13/chorein domain, ATG2 C‐terminal autophagy domain (CAD motif) and ATG2 C‐terminal domain. Position and sequence of the new ATG2 LIR motif that directs GABARAP interaction (orange) and the previously identified WIPI4 interaction motif (YFS; purple box). Approximately 30 amino acids separate these two motifs in human ATG2A and ATG2B. Multiple sequence alignment of multiple species using Jalview highlights the conservation of these regions. Abbreviations for species: Hs—Homo sapiens; Mm—Mus musculus; Rn—Rattus norvegicus; Pt—Pan‐troglodytes; Xt—Xenopus tropicalis Dr—Danio rerio; Dm—*Drosophila melanogaster*; Ce—*Caenorhabditis elegans*; Sp—*Schizosaccharomyces pombe*; Sc—*Saccharomyces cerevisiae*.Myc‐tagged ATG2A wild type (WT) and ATG2A‐mLIR (orange) were co‐expressed with GFP alone, GFP‐LC3B or GFP‐GABARAP in HEK293T cells, lysed and anti‐GFP nanobodies coupled to agarose were used to immunoprecipitate GFP‐tagged proteins. Samples were subjected to Western blotting and probed for the presence of Myc‐ATG2A in immunoprecipitated samples. Anti‐p62/SQSTM1 was used as an internal control for the immunoprecipitated samples.As in (B) but using Myc‐tagged ATG2B‐WT or ATG2B‐mLIR (orange) co‐expressed with GFP alone, GFP‐LC3B or GFP‐GABARAP. Samples were subjected to Western blotting and probed for the presence of Myc‐ATG2B in immunoprecipitated samples. Anti‐p62/SQSTM1 was used as an internal control for the immunoprecipitated samples.HA‐tagged ATG2A‐WT, ATG2A‐mLIR (orange) and ATG2A‐mYFS (YFS/AAA; purple) were stably expressed in U2OS ATG2A/B double‐knockout cells using retrovirus transduction. Cells were transfected with GFP‐GABARAP and 24 h later grown in complete medium (CM) or starved for 2 h (EBSS). Cells were lysed, and anti‐HA beads were used to immunoprecipitate HA‐tagged ATG2A and processed for Western blot. Blots were then probed with antibodies against HA‐tag (ATG2A), anti‐WIPI4 and anti‐GFP for the presence/absence in immunoprecipitated samples. All blots are representative of at least *n* = 3 independent experiments.Quantification of WIPI4 and GFP‐GABARAP‐ii co‐precipitation with HA‐ATG2A from (D). Bands were normalized against HA‐ATG2A immunoprecipitate and expressed as a fold change compared with HA‐ATG2A‐WT (complete media; CM). Each symbol represents an independent experiment (*n* = 3) and error bars mean ± SD.ATG2A/B double‐knockout cells (DKO) stably expressing HA‐tagged ATG2A‐WT, ATG2A‐mLIR (orange) or ATG2A‐mYFS (YFS/AAA; purple) were transfected with GFP‐GABARAP and 24 h later grown in complete medium (CM) or starved for 2 h (EBSS). Cells were lysed, and GFP‐GABARAP was immunoprecipitated using anti‐GFP‐TRAP beads. Samples were processed for Western blot and probed with anti‐HA‐tag (ATG2A), anti‐WIPI4 and anti‐GFP for their presence/absence in immunoprecipitated samples. All blots are representative of at least *n* = 3 independent experiments.Quantification of HA‐ATG2A and WIPI4 co‐precipitation with GFP‐GABARAP from (F). Results were expressed as a fold change compared with HA‐ATG2A‐WT (complete media; CM). Each symbol represents an independent experiment (*n* = 3) with error bars mean ± SD.GFP alone, GFP‐GABARAP or GFP‐GABARAP with increasing concentrations of mCherry‐WIPI4 was expressed in HEK293T cells for 24 h, lysed and GFP‐TRAP beads used to immunoprecipitate GFP alone or GFP‐GABARAP. Samples were then probed with antibodies to detect endogenous ATG2A or ATG2B in immunoprecipitated samples. Blots are representative of *n* = 3 independent experiments.Quantification of ATG2A (green line, round symbols) and ATG2B (grey line and squares) co‐precipitation with GFP‐GABARAP in the presence of increasing concentrations of mCherry‐WIPI4 from (H). Co‐precipitation was normalized to GFP‐GABARAP alone. Line and error bars are mean ± SD of *n* = 3 independent experiments. Source data are available online for this figure.

Direct interaction with Atg8/LC3/GABARAP proteins is mediated through the presence of a LIR (LC3 interaction region) on the target protein 5, 13, 34, 35, 36, 37. ATG2 proteins have previously been shown to be part of the mammalian LC3/GABARAP interactome 38, but no direct link interaction, or consequences, has been shown. We therefore performed an *in silico* analysis of both ATG2A and ATG2B proteins using the iLIR tool 39, as well as manual annotation, to identify potential LIRs that conform to the [E/D/S/T]‐W/F/Y‐X_1_‐X_2_‐L/I/V consensus sequence. We excluded potential LIR sequences present on secondary structures or within domains, as LIR sequences are most frequently found within disordered regions between domains 40. We found that ATG2A contained five, and ATG2B contained six, potential LIRs (Table 1 and Fig EV2A). We then mutated the core sequence of all the potential LIR sequences in both ATG2A and ATG2B to alanine residues (Table 1) and tested the interaction using purified GST (glutathione S‐transferase)‐tagged ATG8 proteins 5. Out of the five potential LIRs present within ATG2A (Fig EV2A–E) and six potential LIRs of ATG2B (Fig EV2A, F and G), only a single, highly conserved functional LIR was present in both ATG2A (LIR#5) and ATG2B (LIR#6; Fig EV2A). Mutation of ATG2A‐LIR#5 (amino acids 1,362–1,365; SDEFCIL; Fig EV2H, mLIR) and ATG2B‐LIR#6 (amino acids 1,491–1,494; NDDFCIL; Fig EV2H, mLIR) reduced ATG2A/B and GST‐ATG8 interaction, compared with WT proteins (Fig EV2H). Overexpression of ATG2A‐LIR (Fig 2B) or ATG2B‐LIR mutants (mLIR; FCIL/AAAA; Fig 2C) with GFP alone, GFP‐LC3B or GFP‐GABARAP abolished co‐precipitation with GFP‐GABARAP, compared with WT proteins. No interaction with GFP only and only weak interaction with LC3B were detected (Fig 2B and C), consistent with previous results (Fig EV1D).

**Table 1:** Potential LC3 interaction region sequences identified in human ATG2A and ATG2B protein sequences

LIR number	Gene	Potential LIR sequence	Amino acid position (WxxL)	Mutant LIR interaction with GST‐LC3/GABARAPs?
1	ATG2A	GTSEPE**YTEI**LT	536–539	+++
2	ATG2A	SLHQST**FSTL**VT	926–929	−
3	ATG2A	GQPGLG**YFCL**EA	981–984	+++
4	ATG2A	HSQLLE**FLDV**LDD	1,092–1,095	+++
5	ATG2A	TLDSDE**FCIL**DAP	1,362–1,365	−
1	ATG2B	SSDDFD**WPRI**VL	845–848	+++
2	ATG2B	LPNKSF**YEKL**YN	954–957	Present on alpha helix
3	ATG2B	PSPVET**FENI**SY	979–982	Not expressed
4	ATG2B	EETLQY**FSTV**DP	1,026–1,029	++
5	ATG2B	INLSRD**YVRV**MD	1,306–1,309	Not expressed
6	ATG2B	PTENDD**FCIL**FAP	1,491–1,494	−

Alignment of the amino acid sequences of ATG2 proteins from multiple species revealed that the ATG2A/2B LIR sequence is highly conserved in multiple vertebrates and invertebrates (Fig 2A, orange box). This includes organisms with a single ATG2 isoform: *Drosophila melanogaster* (DmAtg2), *Caenorhabditis elegans* (CeAtg2), and species with two ATG2 isoforms: *Danio* rerio (DrAtg2a/DrAtg2a) and *Xenopus* tropicalis (XtAtg2a/XtAtg2b). However, the LIR does not appear to be present in *Saccharomyces cerevisiae or Saccharomyces pombe*, indicating a potential divergence in Atg2 function (Fig 2A, orange box). Taken together, both ATG2A and ATG2B contain a single, highly conserved LIR motif that preferentially interacts with the GABARAP and GABARAP‐L1 proteins.

Since the first LC3 interaction region was identified in the prototypical autophagy receptor protein, p62/SQSTM1 (Sequestosome‐1) 10, the number of functional LIR‐containing proteins identified to date has grown considerably. The interaction between mammalian ATG8s and LIR‐containing proteins serves to control all aspects of the autophagy pathway, from cargo selection to formation, transport and fusion of the autophagosome. Not only are these interaction sequences present in mammalian, plant, fungi and invertebrate species, but they are also present in a number of viral 41 and bacterial 42 proteins, potentially to aid pathogen survival and subversion of the pathway. We have identified a highly conserved LIR within both ATG2A and ATG2B that differ only in a few amino acids both N‐and C‐terminal of the core LIR sequence (FCIL; Fig 2A). This raises an ongoing question as to how specificity within the system is achieved, particularly in mammalian systems that are complicated by the expression of six LC3/GABARAP isoforms. We, and others 13, 43, 44, have attempted to decipher the code that dictates whether a protein with a particular LIR sequence will preferentially interact with LC3 over GABARAP. Interestingly, ATG2A and ATG2B do not conform to the recently identified GABARAP interaction motif consensus sequence (W/F‐I/V‐X‐I/V) 13 despite preferring GABARAP and GABARAP‐L1 over LC3 in co‐immunoprecipitation from cells (Figs EV1D, 1E and 2B and C). Surprisingly, both ATG2A and ATG2B can also interact with LC3A (Fig EV1D); however, we have been unable to confirm an endogenous interaction. The high degree of conservation of the ATG2A/B LIR sequence throughout vertebrates and invertebrates (Fig 2A) potentially indicates a conserved function, even in species with only a single ATG2 isoform, such as *D. melanogaster* and *C. elegans*. Therefore, understanding the role of ATG2‐LC3/GABARAP interaction during autophagy will provide insights into ATG2s mechanism of function in multiple species.

### WIPI4 can negatively regulate the ATG2A‐GABARAP interaction

Intriguingly, the recently described WIPI4 interaction region that contains an essential Y/HFS motif 30, 31 is approximately 30‐amino acid C‐terminal of the newly identified LIR sequence in both ATG2A and ATG2B (Fig 2A, purple box). Therefore, we wanted to test whether the interaction between ATG2, GABARAP and WIPI4 was co‐dependent, or whether they represented independent interactions. The YFS motif found in ATG2A (amino acids 1,395–1,397; YFS/AAA; mYFS) was mutated, and HA‐tagged ATG2A‐WT, ATG2A‐mLIR or ATG2A‐mYFS variants were stably expressed in ATG2A/B double‐knockout cells (DKO; Fig EV2I), immunoprecipitated from cell lysates and probed for the presence of GFP‐tagged GABARAP and endogenous WIPI4. ATG2A‐WT co‐precipitated both WIPI4 and GFP‐GABARAP under complete media (CM) and starvation conditions (Fig 2D and quantified in 2E). ATG2A‐LIR mutant failed to co‐precipitate GFP‐GABARAP, whereas the ATG2A(mLIR)‐WIPI4 interaction was slightly enhanced (Fig 2D and quantified in 2E; mLIR). Conversely, mutation of the ATG2A‐YFS motif resulted in the loss of WIPI4 interaction and increased interaction with lipidated GABARAP (GABARAP‐ii; Fig 2D and quantified in 2E; mYFS). Immunoprecipitation of GFP‐GABARAP resulted in WIPI4 co‐precipitation only in the presence of ATG2A‐WT, indicating that WIPI4 interacts only with ATG2A and not GABARAP directly (Fig 2F and quantified in 2G). Next, we overexpressed GFP alone, GFP‐GABARAP alone or GFP‐GABARAP with increasing concentrations of mCherry‐WIPI4, immunoprecipitated the GFP‐tag and asked whether we could outcompete the binding of GABARAP to ATG2A/2B. Increasing expression of mCherry‐WIPI4 resulted in a steady decline in endogenous ATG2A/ATG2B immunoprecipitation with GFP‐GABARAP (Fig 2H and quantified in 2I). Interestingly, increasing mCherry‐GABARAP expression had little effect on GFP‐WIPI4 precipitation of ATG2A/ATG2B (Fig EV2J and quantified in Fig EV2K). Thus, within a 30‐amino acid stretch mammalian ATG2 proteins are two distinct interaction motifs that can potentially regulate ATG2 function at the growing phagophore membrane.

### ATG2A‐LIR mutant but not WIPI4 mutant is sufficient to block autophagy

**Figure 3:**
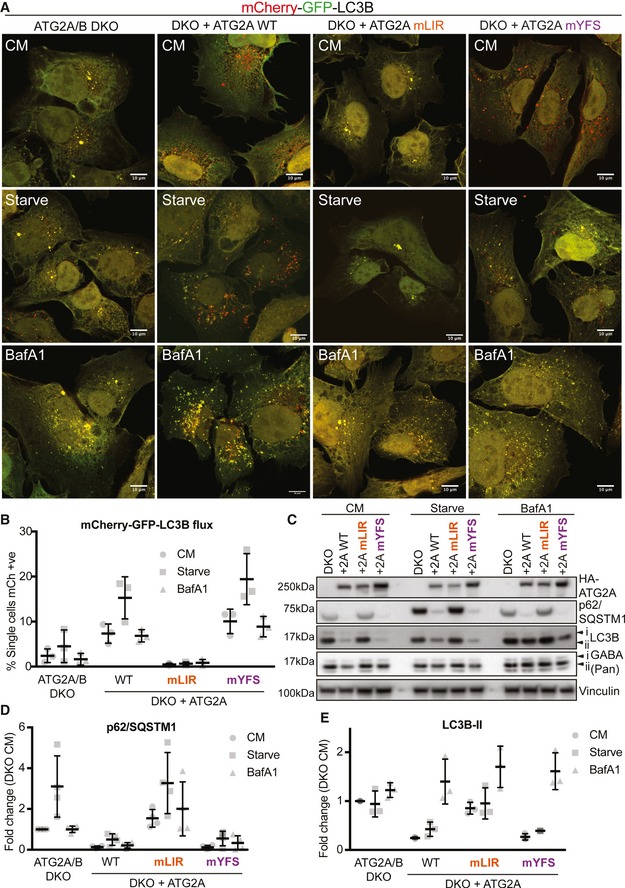
ATG2A LIR domain is essential for autophagy flux AU2OS ATG2A/B double‐knockout (DKO) CRISPR/Cas9 cells stably expressing tandem‐tagged LC3B (mCherry‐GFP‐LC3B) were retrovirally transduced to express vector, or HA‐tagged ATG2A‐WT, ATG2A‐mLIR (FCIL/AAAA) or ATG2A‐mYFS (YFS/AAA). Cells were grown in complete medium (CM) or starved for 2 h (EBSS) or treated with CM plus bafilomycin A1 (200 nM, 4 h), fixed and analysed by confocal microscopy. Merged images of GFP (green) and mCherry (red) channels show the presence of autophagosomes/phagophores (GFP‐ and mCherry‐positive, yellow puncta) or autolysosomes (mCherry only, red puncta) Scale bar 10 μm. Images are representative of *n* = 3 independent experiments.BQuantification of (A) using flow cytometry of measuring GFP and mCherry fluorescence. Cells were gated based on GFP and mCherry fluorescence and % mCherry‐positive cells gated used as an indication of autolysosome formation due to GFP quenching. Each symbol represents 1 independent experiment with 10,000 cells analysed per condition. A total of *n* = 3 independent experiments were performed, and horizontal bar indicates mean ± SD.CU2OS ATG2A/B DKO cells reconstituted with vector only, HA‐ATG2A‐WT, HA‐ATG2A‐mLIR and HA‐ATG2A‐mYFS were stimulated with complete medium (CM), 2‐h starvation (EBSS) or 4‐h bafilomycin A1 (BafA1, 200 nM), lysed in total cell lysis buffer and subjected to Western blot analysis. Blots were probed for the presence of HA‐tag (ATG2A), p62/SQSTM1, LC3B, pan‐GABARAP (GABARAP, GABARAP‐L1 and GABARAP‐L2) and vinculin (loading control).D, Ep62/SQSTM1 (D) and LC3B‐II (E) levels were normalized to loading control and quantified as fold change of DKO proteins levels. Each symbol represents an independent experiment. Quantification of at least *n* = 3 independent experiments is shown. Horizontal bar represents mean ± SD.

ATG2A can simultaneously interact with both GABARAP and WIPI4 (Fig 2D–F). Interestingly, previous reports that identified the WIPI4 interaction region on ATG2 30, 31 and the ATG2 interaction site of WIPI4 29 did not address the role of these interaction mutants during autophagy flux. Considering the close proximity of both GABARAP and WIPI4 interaction motifs on ATG2A, we wanted to dissect the individual roles of ATG2A‐GABARAP and ATG2A‐WIPI4 interactions during autophagy. Stable expression of the tandem‐tagged LC3B autophagy reporter (mCherry‐GFP‐LC3B; 45) in ATG2A/B DKO cells was used to assess LC3B transition from autophagosomes (GFP+ve/mCherry+ve) to autolysosomes (GFP−ve/mCherry+ve) due to GFP quenching at low pH 45. Using confocal imaging and flow cytometry to quantify, tandem‐tagged LC3B puncta in ATG2A/2B DKO cells under complete medium (CM) or starvation conditions were both GFP‐ and mCherry‐positive (Fig 3A and quantified in 3B). This indicated that the ATG2A/B DKO U2OS cells had impaired autophagy flux, consistent with previous work 46. Stable reconstitution of tandem‐LC3B expressing ATG2A/B DKO cells with ATG2A‐WT resulted in more mCherry‐only‐positive cells/puncta in CM conditions that were increased upon starvation and was nullified using bafilomycin A1 (to prevent lysosome acidification and quenching of GFP signal) (Fig 3A and quantified in 3B). Surprisingly, expression of ATG2A‐mLIR resulted in a complete lack of mCherry‐only puncta/cells, resembling the ATG2A/B DKO cells, and unexpectedly, the ATG2A‐mYFS (WIPI4 mutant) was able to fully restore autophagy flux similar to ATG2A‐WT (Fig 3A and quantified in 3B).

**Figure EV3:**
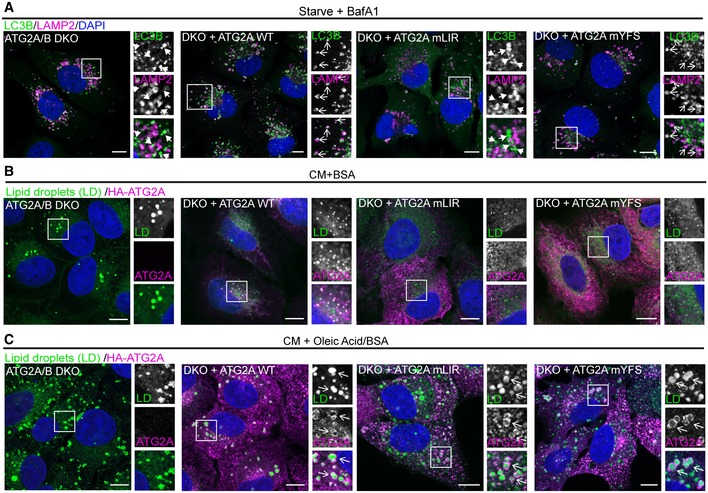
ATG2A‐WT and mutants effectively localize to lipid droplets. Related to Fig 3 AU2OS ATG2A/B DKO cells reconstituted with vector only, HA‐ATG2A‐WT, HA‐ATG2A‐mLIR and HA‐ATG2A‐mYFS were stimulated starvation (EBSS) plus bafilomycin A1 (BafA1, 200 nM) for 4 h to stimulate autophagosome generation and prevent their degradation in the lysosome. Cells were fixed and immune‐stained for LC3B (green) and LAMP2 (magenta) to visualize lysosomes. DAPI was included (blue) to mark the DNA/nucleus. Closed arrows (ATG2A/B DKO and DKO + ATG2A mLIR) highlight aggregate structures. Open arrows (DKO + ATG2A‐WT and ATG2A‐mYFS) highlight LAMP2/LC3B‐positive vesicles. Scale bar 10 μm.B, CU2OS ATG2A/B DKO cells reconstituted with vector only, HA‐ATG2A‐WT, HA‐ATG2A‐mLIR and HA‐ATG2A‐mYFS were stimulated with either 2% BSA only (B) or 2% BSA plus 500 μM oleic acid (C) for 16 h prior to fixation in 4% PFA. Cells were permeabilized using saponin and stained with anti‐HA (ATG2A; magenta) and 5 μM BODIPY 493/503 to visualize lipid droplets (green; LDs). DAPI was included (blue) to mark the DNA/nucleus. Open arrows highlight ATG2A‐positive lipid droplets. All images are representative of at least *n* = 3 independent experiments. Scale bar 10 μm.

Next, we analysed the effect of ATG2A‐WT, ATG2A‐mLIR and ATG2A‐mYFS expression on both p62/SQSTM1, LC3B and GABARAP protein levels, as these are autophagy substrates and are good indicators of flux 47. Stable expression of HA‐tagged ATG2A‐WT in ATG2A/2B DKO cells resulted in decreased p62/SQSTM1 and LC3B‐II levels, compared with DKO alone, indicating rescue of the pathway and restoration of autophagy flux (Fig 3C–E, WT lane). Consistent with the tandem‐tagged LC3B reporter assay (Fig 3A and B), expression of ATG2A‐mYFS was able to fully restore autophagy flux under nutrient‐rich (CM, complete medium), starvation and bafilomycin treatment (Fig 3C–E). However, GABARAP proteins did not show clear changes in flux as LC3B and p62, indicating that these may not be the best measure of autophagy flux (Fig 3C). Excitingly, the expression of ATG2A‐mLIR failed to rescue the defect in p62/SQSTM1 and LC3B‐II (Fig 3C–E). In DKO plus ATG2A‐WT and DKO plus ATG2A‐mYFS expressing cells, LC3B was present within LAMP2‐positive vesicles (lysosomes) after starvation plus bafilomycin A1 treatment, to induce autophagosome formation but halt their degradation (Fig EV3A, open arrows). In stark contrast, LC3B was observed juxtaposed to LAMP2 vesicles in both ATG2A/2B DKO and DKO plus ATG2A‐mLIR (Fig EV3A, closed arrows), indicating an autophagosome maturation defect and consistent with the tandem‐LC3B reporter assay (Fig 3A and B). One aspect of mammalian ATG2 function is the regulation of the size and distribution of lipid droplets (LDs) 33. ATG2A localizes to the limiting membrane of LDs 33, 46. Importantly, both HA‐tagged ATG2A‐mLIR and ATG2A‐mYFS, as well as HA‐ATG2A‐WT, are able to localize to lipid droplets induced by oleate, a fatty acid supplement that induces the accumulation of neutral lipids into LDs (Fig EV3B and C). Therefore, disruption of either the ATG2A‐GABARAP or ATG2A‐WIPI4 interaction does not affect ATG2A localization to LDs. Taken together, our data show that mutation of a conserved GABARAP/GABARAP‐L1 interaction motif on ATG2A fails to restore the autophagy defect of ATG2A/ATG2B double‐knockout cells, whereas the interaction with WIPI4 is dispensable for autophagy flux.

### Mutation of the ATG2A‐GABARAP interaction impairs phagophore closure

**Figure 4:**
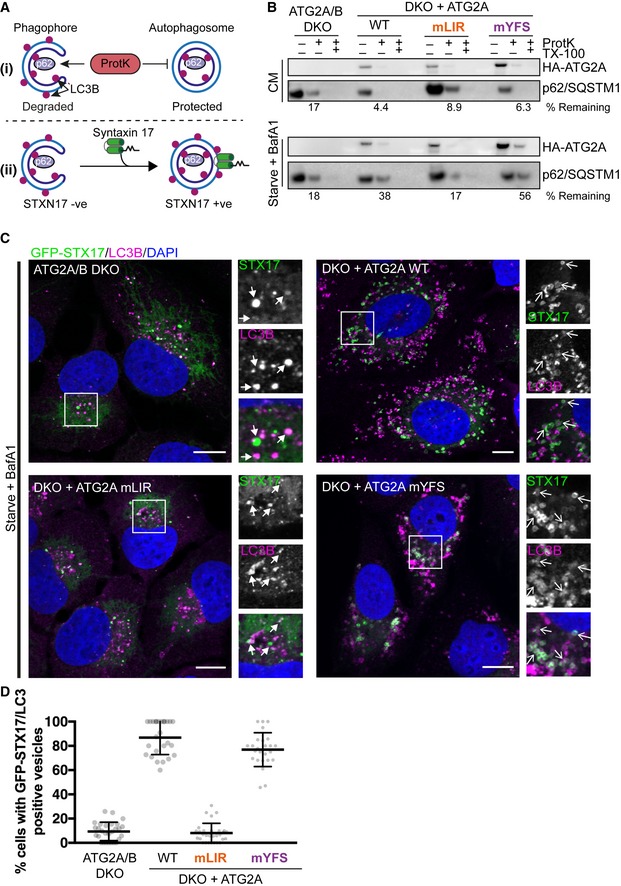
Mutation of ATG2A LIR prevents phagophore closure Graphical representation of proteinase K assay (i) showing protection of p62/SQSTM1 inside sealed autophagosomes or proteinase K‐sensitive p62 present within open phagophores. (ii) Graphical representation of sytnatxin17 (STX17) translocation to completed autophagosomes and not phagophores. Autophagosomes are identified as being both LC3B‐ and STX17‐positive vesicles.U2OS ATG2A/B DKO cells reconstituted with vector only, HA‐ATG2A‐WT, HA‐ATG2A‐mLIR and HA‐ATG2A‐mYFS were stimulated with complete medium (CM), 4‐h starvation (EBSS) plus bafilomycin A1 (BafA1, 200 nM) treatment. Cells were centrifuged and resuspended in PBS digitonin, spun, washed and the membrane fractions incubated with proteinase K with and without 0.1% Triton X‐100. Samples were then subjected to Western blotting using anti‐p62/SQSTM1 and anti‐HA (ATG2A) antibodies. Percentage p62/SQSTM1 remaining was calculated using densitometry analysis. Blots are representative of *n* = 3 independent experiments.U2OS ATG2A/B DKO cells reconstituted with vector only, HA‐ATG2A‐WT, HA‐ATG2A‐mLIR and HA‐ATG2A‐mYFS and stably expressing GFP‐Syntaxin 17 (STX17) were stimulated starvation (EBSS) plus bafilomycin A1 (BafA1, 200 nM) for 4 h to stimulate autophagosome generation and prevent their degradation in the lysosome. Cells were fixed and immune‐stained for LC3B (magenta). DAPI was included (blue) to mark the DNA/nucleus. Confocal analysis of LC3B and GFP‐STX17 (green) localization was performed. Closed arrows (ATG2A/B DKO and DKO + ATG2A mLIR) highlight aggregate structures. Open arrows (DKO + ATG2A‐WT and ATG2A‐mYFS) highlight STX17/LC3B‐positive vesicles. Scale bar 10 μm.Quantification of (C) expressed as a percentage of cells with STX17/LC3B‐positive vesicles. Each symbol represents a single field of cells with 5–10 cells per field. A total of 300–600 cells were analysed over *n* = 3 independent experiments. Data are shown as mean ± SD.

The mammalian ATG2 proteins have been suggested to function at either the initial formation of phagophores 32, 33 or the transition from phagophore to autophagosome, the closure step 48. Therefore, in order to address the functional significance of the ATG2 interaction with GABARAP, we used two assays to distinguish between phagophores and autophagosomes—a proteinase K protection assay (Fig 4A(i)) and Syntaxin 17 (STX17) translocation (Fig 4A(ii)). Firstly, using the proteinase K limited proteolysis assay, which degrades proteins not protected within a membrane compartment (Fig 4A (i)), we tested whether the expression of ATG2A‐LIR mutant resulted in defective phagophore closure. ATG2A/B DKO cells and DKO cells reconstituted with ATG2A‐WT, ATG2A‐mLIR or ATG2A‐mYFS (Fig 4B) were left in CM or starved for 4 h in the presence of bafilomycin A1 (Starve+BafA1) to accumulate autophagosomes. Cells were then permeabilized using digitonin and incubated in buffer only, proteinase K or proteinase K plus Triton X‐100 (to permeabilize membranes). Under CM conditions, the majority of the autophagy substrate p62 was degraded in all samples (Fig 4B, upper panel). After starvation plus bafilomycin A1 treatment, DKO cells reconstituted with ATG2A‐WT and ATG2A‐mYFS showed a large proportion of p62 resistant to proteinase K degradation (Fig 4B, lower panel; 38 and 56%, respectively). However, p62 in both DKO and DKO plus ATG2A‐mLIR cells was sensitive to proteinase K digestion (Fig 4B, lower panel; 18 and 17%, respectively), which is indicative of immature/unsealed autophagosomes.

Next, STX17 translocation to LC3B‐positive vesicles in the ATG2A‐WT and mutant‐expressing cells was tested. STX17 translocates from the ER to fully formed autophagosomes, but not phagophores, prior to their fusion with the lysosome (Fig 4A (ii)) 49, 50, 51. Stable expression of GFP‐Syntaxin 17 in the reconstituted ATG2A/B DKO cells revealed that STX17 can efficiently localize to, and surround, LC3B‐positive structures in both ATG2A‐WT and ATG2A‐mYFS expressing cells after starvation plus bafilomycin A1 treatment (Fig 4C, open arrows and quantified in Fig 4D). Conversely, in ATG2A/B DKO and DKOs plus ATG2A‐mLIR cells, GFP‐STX17 localized mainly to ER and punctate structures with few GFP‐STX17+ve/LC3B+ve vesicles observed (Fig 4C, closed arrows and quantified in Fig 4D). Taken together, our data suggest that a conserved GABARAP interaction motif in both mammalian ATG2A is essential for phagophore formation and/or closure and surprisingly that the WIPI4 interaction is dispensable for this function during starvation‐induced autophagy.

### ATG2A‐LIR is essential for autophagosome formation

**Figure EV4:**
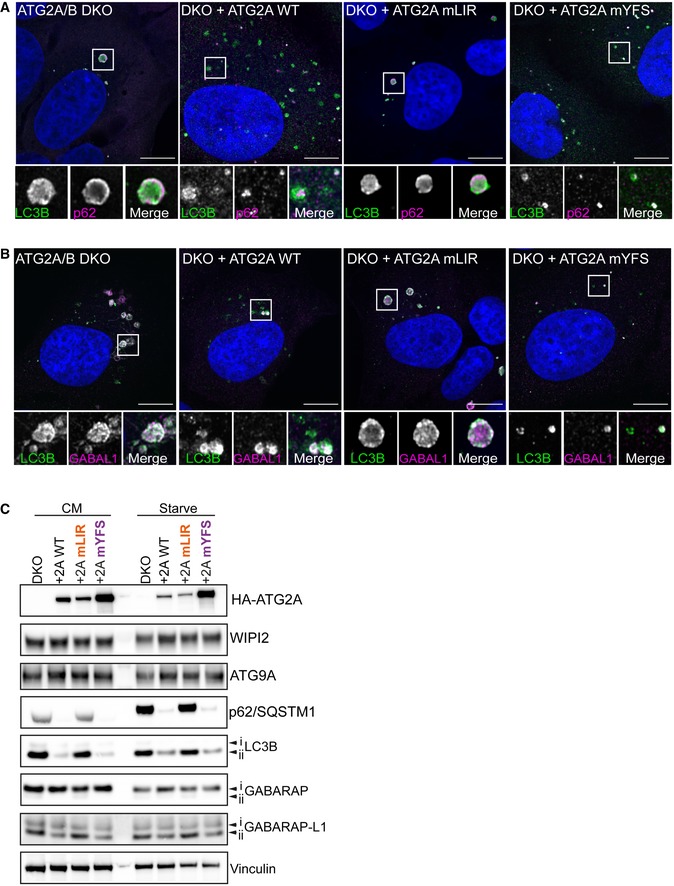
ATG2A‐mLIR causes accumulation of early autophagy markers. Related to Fig 5 A, BU2OS ATG2A/B DKO cells reconstituted with vector only, HA‐ATG2A‐WT, HA‐ATG2A‐mLIR and HA‐ATG2A‐mYFS were stimulated starvation (EBSS) 2 h to stimulate autophagosome generation. Cells were then fixed and immune‐stained for LC3B (green), p62/SQSTM1 (magenta) (A) or GABARAP‐L1 (magenta) (B) and DAPI (blue; DNA/nucleus). Images were taken on a Zeiss 880 AiryScan super‐resolution confocal microscope. All images are representative of at least *n* = 3 independent experiments. Scale bar 10 μm.CTotal cell lysates of ATG2A/B double‐knockout cells alone or reconstituted with HA‐tagged ATG2A‐WT, ATG2A‐mLIR (FCIL/AAAA) or ATG2A‐mYFS (YFS/AAA) and left in complete media (CM) or starved for 2 h (EBSS). Cells were lysed and blotted for autophagy marker proteins p62/SQSTM1, LC3B, ATG9A, WIPI2, GABARAP and GABARAP‐L1. Anti‐HA for ATG2A expression and vinculin were used as a loading control.

**Figure 5:**
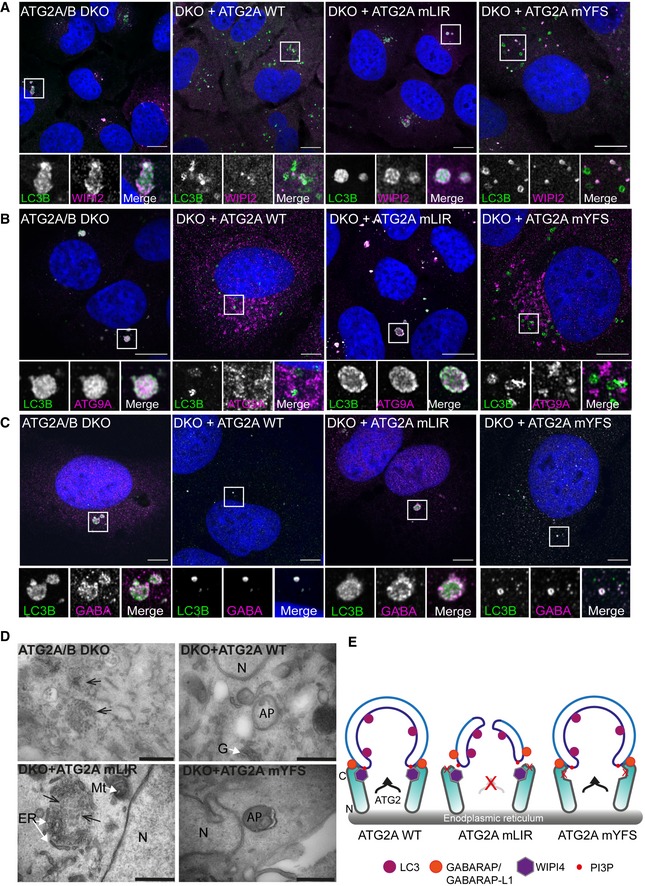
ATG2A‐LIR is essential for phagophore formation A–CU2OS ATG2A/B double‐knockout (DKO) cells or DKO reconstituted with ATG2A‐WT, ATG2A‐mLIR (FCIL/AAAA) or ATG2A‐mYFS (YFS/AAA) were starved for 2 h, fixed and stained for LC3B (green) or WIPI2 (magenta), (B) ATG9A (magenta) or (C) GABARAP (Magenta) and imaged using a Zeiss 880 AiryScan super‐resolution confocal microscope. Images are representatives of *n* = 3 independent experiments. Scale bar 10 μm.DTransmission electron micrographs of ATG2A/B DKO cells (upper left), DKO + ATG2A‐WT (upper right), DKO+ ATG2A‐mLIR (lower left) and DKO+ATG2A‐mYFS (lower right) starved (EBSS) for 2 h. Clustered small vesicles (open arrowheads; DKO and DKO‐mLIR) and autophagosomes (closed arrow heads, DKO+ATG2A‐WT and DKO+ATG2A‐mYFS) indicated. Images are representative of *n* = 3 independent experiments. Scale bar 500 nm. ER = endoplasmic reticulum; *N* = nucleus; Mt = mitochondria; G = Golgi; AP = autophagosome.EModel of ATG2 function based on the current knowledge. ATG2A localizes to ER membranes and facilitates lipid transfer from the ER to the growing phagophore. ATG2 interaction with GABARAP/GABARAP‐L1 is essential for anchoring ATG2 to growing phagophore and mutation of the GABARAP interaction region results in the formation of immature phagophores.

To gain a better understanding of the impact the ATG2A‐LIR mutation has on phagophore formation and/or closure, we used super‐resolution confocal microscopy to assess autophagosome formation under starvation conditions. In ATG2A/2B DKO and DKO cells expressing ATG2A‐mLIR, the cells exhibited large LC3B‐positive/p62‐positive structures (Fig EV4A). Moreover, these structures showed WIPI2 staining throughout (Fig 5A) and accumulated ATG9A (Fig 2B), GABARAP (Fig 5C) and GABARAP‐L1 (Fig EV4B). Interestingly, despite their accumulation, little difference was observed in the total protein levels of ATG9A, WIPI2 and slight changes in lipidated GABARAP and GABARAP‐L1, compared with p62 and LC3B (Fig EV4C). In contrast, ATG2A/B DKO cells reconstituted with ATG2A‐WT or ATG2A‐mYFS resulted in vesicular LC3 and punctate p62/SQSTM1 structures (Fig EV4A), punctate WIPI2 (Fig 2A), juxtanuclear ATG9A localization (Fig 2B) and vesicular GABARAP (Fig 2A) and GABARAP‐L1 (Fig EV4B) consistent with restoration of efficient autophagosome biogenesis and autophagy flux.

In cells expressing ATG2A‐mLIR, LC3B is lipidated (LC3B‐ii; Figs 3C and EV4C), early phagophore‐associated proteins are present (ATG9A, Fig 5B), GABARAP and GABARAP‐L1 are also associated (Figs 5C and EV4B, respectively) and the membranes contain PI3P (inferred by the presence of WIPI2, Fig 5A). This indicates that the observed structures (Figs 5A–C and EV4A and B) are most likely immature phagophore membranes. Indeed, the structures observed in ATG2A/DKO and DKO+ATG2A‐mLIR expressing cells resemble small, clustered vesicles as shown for GABARAP‐L1 (magenta) and LC3B (green) in Movie EV1 (DKO) and Movie EV2 (DKO+ATG2A‐mLIR). Lastly, we used transmission electron microscopy to ascertain the nature of the membrane clusters we observed (Figs 5A–C and EV4A and B). Notably, in both ATG2A/B DKO and DKO+ATG2A‐mLIR expressing cells, we observed small, clustered vesicles interlaced with endoplasmic reticulum that were not evident in ATG2A‐WT or ATG2A‐mYFS expressing cells that contained double‐membraned autophagosomes/phagophores (Fig 5D). Taken together, we show that an ATG2A‐GABARAP/GABARAP‐L1 interaction is essential for efficient phagophore formation and eventual closure.

The formation of the autophagosome and its subsequent trafficking and fusion with the lysosome is a tightly controlled pathway with a number of essential components that allows it to progress in an orderly fashion. This enables the cell to liberate amino acid and lipid stores during periods of stress, target and remove intracellular pathogens or remove cytotoxic protein aggregates from the cell. Critical to this process is the ability of the cell to form a double‐membraned phagophore that grows, surrounds and isolates the material to be removed. Despite recent advances in our knowledge, the mechanisms involved in phagophore closure are poorly understood. Recent work has shown that the ESCRT‐III component CHMP2A regulates the separation of inner and outer phagophore membranes 23, and more recently, VPS37A is essential for phagophore closure 52. In addition to the ESCRT‐III machinery, TRAPPC11, a member of TRAPP complexes involved in membrane trafficking, has been shown to recruit ATG2B‐WIPI4 to phagophores in an ATG9A‐dependent manner 53. The depletion of TRAPC11 results in a phenotype similar to that of ATG2A/B DKO and ATG2A‐mLIR 53. The mammalian ATG2 proteins, ATG2A and ATG2B, have been shown to be essential for phagophore formation and closure 32, 33, 46, and depletion of WIPI4, a constitutive interaction partner of mammalian ATG2s, also negatively impacts on phagophore closure 29. Herein, we have described a hitherto unidentified GABARAP/GABARAP‐L1 interaction region on both ATG2A and ATG2B that is essential for phagophore formation.

These results shed new light on the role of ATG2 during autophagosome biogenesis, and in particular, the interactions that are necessary for this process. Perhaps most surprisingly was the effect, or rather lack thereof, that the ATG2A‐WIPI4 interaction mutant had on phagophore closure and autophagy flux. From yeast to fruit flies to humans, the ATG2‐ATG18 (WIPI) interaction is highly conserved. In yeast, Atg2‐Atg18 interaction occurs independently of Atg18 binding PI3P 26, much like the ATG2A/B interaction with WIPI4 29, 30, 31. Yeast Atg2 has been shown to contain both N‐ and C‐terminal membrane binding domains that help tether Atg2 to membrane contact sites 25, and yeast Atg9‐Atg2‐Atg18 complex is important to establish phagophore‐ER contact sites for phagophore expansion 54. Human ATG2A has several domains that determine its ability to localize to membranes. Firstly, ATG2 has an N‐terminal membrane binding region that is essential for autophagosome formation 46 that has now been shown to be a lipid transport domain 55, 56. This N‐terminal lipid transport domain is thought to be essential for the transport of PE and phosphatidylserine from the ER/omegasome to the growing phagophore 55, 56, potentially at mitochondrial‐ER contact sites 57. This domain is homologous to the Vps13 lipid transport domain involved in organelle–organelle contact sites 58. The second lipid interaction region of ATG2A is an amphipathic helix (AH; aa1,750–1,767), which is essential for ATG2 localization to both lipid droplets and isolation membranes and is essential for autophagy flux 46. In addition, ATG2 has a C‐terminal region (aa1,830–1,938 HsATG2A) that is involved in localization to lipid droplets but is dispensable for autophagy 46. This raises an interesting question as to the role of the ATG2‐WIPI4 interaction, as this was previously thought to be involved in ATG2 autophagy function. However, we have shown that ATG2‐WIPI4 is dispensable for autophagosome formation and autophagy flux. Given that the ATG2A‐LIR mutant we identified has impaired autophagy flux (Fig 3) but can still localize to lipid droplets (Fig EV3B and C), we suggest that both the ATG2‐AH and the ATG2‐LIR are essential to define the target membrane, allowing tethering and lipid transfer and driving efficient phagophore formation and autophagosome maturation (Fig 5E).

## Materials and Methods

### Antibodies

The antibodies used in this study are as follows: anti‐GFP (Santa Cruz, clone B‐2, sc9996), anti‐FlagM2 (Sigma, F3165), anti‐p62 (MBL, M162‐3), anti‐LC3B (clone 5F10 Nanotools, 0231‐100/LC3‐5F10) and anti‐GABARAP (Abcam, ab109364), and anti‐ATG16L1 (MBL, PM040), anti‐ATG9A (Abcam, ab108338), anti‐WIPI2 and anti‐WIPI4 were kind gifts from Prof. Sharon Tooze, LC3A clone D50G8 (CST, #4599), anti‐LC3C (D1R8V; CST # 14723), anti‐pan‐GABARAP (GABARAP/GABARAP‐L1/GABARAP‐L2) (Abcam, ab109364), anti‐GABARAP (IF, Western blotting; Abgent, AP1821a), anti‐GATE‐16 (MBL, PM038), anti‐GABARAP‐L1 (WB, IF, Proteintech, 11010‐1‐AP), anti‐vinculin (Sigma, V9131‐100UL), anti‐ATG2A (Proteintech, 23226‐1‐AP) and ATG2B (Proteintech, 25155‐1‐AP), LAMP‐2 (DSHB, clone H4B4) and c‐Myc (DSHB, clone 9E10).

### Cell culture and reagents

HEK293T and U2OS or U2OS ATG2A/B double‐knockout cells were maintained in Dulbecco's modified Eagle's medium (DMEM; Invitrogen 10313021) supplemented with 10% foetal bovine serum (FBS), 5 U/ml penicillin and 50 μg/ml streptomycin, 1 mM l‐glutamine and 1% sodium pyruvate. For starvation in nutrient‐depleted medium, the cells were incubated 2 h in Earle's balanced salt solution (EBSS; Gibco, 24010‐043). Bafilomycin A1 (BafA1; Enzo, BML‐CM110‐0100) was used at 200 nM. ATG2A/2B DKO cells were stably transfected by retroviral transduction of pMSCV‐Flag‐HA (iTAP) vectors. Briefly, HEK293T cells were transfected with iTAP vectors with pCG‐GagPol and pCG‐VSVG (retrovirus) packaging vectors. Virus‐containing media was harvested 48 h post‐transfection, centrifuged at 300× *g*, passed through a 0.45‐μm filter and added to ATG2A/B DKO cells in the presence of 10 μg/ml polybrene (Sigma, H9268‐5G). Transduced cells were selected by addition of 1 μg/ml puromycin (iTAP and GFP‐STX17) 48 h after addition of viral media.

### CRISPR/Cas‐9 gene editing

CRISPR/Cas9‐mediated deletion of ATG2A (NM_015104.3) and ATG2B (NM_018036.7) in osteosarcoma cells (U2OS) was performed by using the Cas9 D10A “nickase” mutant, and paired gRNA approach 59 was used to target exon 1 of both ATG2A (5′‐CCATGGTCAAACTGTGTGAAAGA‐3′ and 5′‐TACTTGCTGCACCACTACTTAGG‐3′) and ATG2B (5′‐CCGTTTTCGGAGTCCATCAAGAA‐3′ and 5′‐CCTGCCGGTACCTCCTGCAGAGG‐3′). ATG2A‐ and ATG2B‐targeting gRNAs were transfected into 1 × 10^6^ U2OS cells followed by selection with 1 μg/ml puromycin for 48 h, re‐transfection, recovery (in puromycin‐free media) and single‐cell sorting to isolate clone candidates with the gene deletion.

Endogenous GFP‐tagged ATG2A knock‐ins were generated using a modified “nickase” strategy (as above). Optimal sgRNA pairs were identified and chosen on the basis of being as close as possible to the point of GFP insertion while having a low combined off‐targeting score (ATG2A‐sgRNA1: 5′‐GTCAAACTGTGTGAAAGAGC‐3′ and sgRNA2: 5′‐AGATGTCACGATGGCTGTGGC‐3′). Complementary oligos with BbsI compatible overhangs were designed for each, and these dsDNA guide inserts ligated into BbsI‐digested target vectors; the antisense guide (sgRNA2) was cloned onto the spCas9 D10A expressing pX335 vector (Addgene plasmid no. 42335) and the sense guides (sgRNA1) into the puromycin‐selectable pBABED P U6 plasmid (Dundee‐modified version of pBABE‐puro plasmid). A donor construct consisting of GFP flanked by approximately 500‐bp homology arms was synthesized by GeneArt (Life Technologies); each donor was engineered to contain sufficient silent mutations to prevent recognition and cleavage by Cas9 nuclease. Both sgRNA and donor constructs were transfected into U2OS cells, selected in 1 μg/ml puromycin for 48 h, re‐transfected and allowed to recover in puromycin‐free complete media. When confluent, cells were single‐cell‐sorted for GFP‐positive populations and homozygous clones selected for further analysis.

### Western blot and immunoprecipitation

Cells (HEK293T, U2OS) were lysed in NP‐40 lysis buffer (50 mM Tris, pH7.5, 120 mM NaCl, 1% NP‐40) supplemented with Complete^®^ protease inhibitor (Roche) and phosphatase inhibitor cocktail (Roche). Lysates were passed through a 27G needle, centrifuged at 21,000× *g* and incubated with either anti‐GFP agarose (Chromotek, gta‐20) or anti‐HA agarose (Sigma, A2095), washed three times in lysis buffer and subjected to SDS–PAGE and Western blot. For total cell lysis (TCL), cells were lysed in 50 mM Tris, pH 7.5, 150 mM NaCl, 1 mM MgCl_2_, 1% SDS. TCL buffer was supplemented with Complete^®^ protease inhibitor (Roche), phosphatase inhibitor cocktail (Roche) and Benzonase (VWR/Fisher Scientific) at 1 μl per ml buffer. Samples were boiled in 3× Laemelli buffer prior to SDS–PAGE. Unless otherwise stated, NuPAGE™ 4–12% Bis–Tris gradient gels (Invitrogen) were used. Gels were transferred onto activated PVDF membranes (Immobilon‐Psq, 0.2 μm, Merck) prior to blocking and incubation with the indicated primary antibodies.

### Autophagy flux by flow cytometry assay

U2OS ATG2A/2B DKO cells were transfected with mCherry‐EGFP‐LC3B tandem‐tagged reporter construct 45, 60, grown in G418/neomycin selection (800 μg/ml) and single‐cell‐cloned. U2OS‐ATG2A/B DKO‐tandem LC3B cells were then transduced with retrovirus containing iTAP ATG2A constructs and selected for in G418 + 1.5 μg/ml puromycin and stable cells generated. These were treated as indicated, scraped in PBS and fixed in 4% PFA for 15 min, washed and then subjected to flow cytometry analysis. All flow cytometry experiments were carried out at least three times using 10,000 cells per cell line per treatment. The cells were then analysed and sorted on an LSR Fortessa (Becton Dickinson) flow cytometer. Cells were gated according to forward scatter and side scatter, and dead cells were excluded from analysis. GFP fluorescence measured by excitation at 488 nm and emission detected at 530 ± 30 nm and mCherry fluorescence measured by excitation at 561 nm and emission detected at 610 ± 20 nm. Flow data were analysed using FlowJo software.

### Immunofluorescence and confocal microscopy

Cells grown on 18‐mm glass coverslips were treated as described and subsequently fixed in 4% paraformaldehyde/PBS (PFA; Santa Cruz, 30525‐89‐4) for 10 min at room temperature and washed 3× in PBS. Cells were then washed in PBS/0.1% saponin twice and primary antibodies incubated for 1 h at room temperature in 5% BSA/PBS/0.1% saponin. DAPI (Molecular Probes) was added during primary antibody incubation. Coverslips were then washed twice in PBS/0.1% saponin, and secondary antibodies (Invitrogen donkey anti‐mouse, anti‐rabbit, anti‐rat) and Alexa dyes (488, 555 and 647) were used in combination depending on the primary antibody species and incubated in PBS/5% BSA/0.1% saponin. For detection of endogenous GFP‐ATG2A, nanobody boosters towards GFP (anti‐GFP, Atto‐488 Coupled, Chromotek; gba488‐100) were used to enhance the signal. Secondary antibodies were then washed twice in PBS/0.1% saponin, once in PBS and once in ddH_2_O to remove the residual saponin prior to mounting in ProLong Diamond Antifade containing Mowiol (Invitrogen, p36965). Cells were imaged using a Zeiss 710 confocal microscope with a 63× objective lens. Super‐resolution microscopy images were taken on Zeis 880 AiryScan, and image processing was carried out using built in‐software (Zen Software AiryScan Processing). Subsequent image analysis was performed using FIJI (ImageJ) 61.

### Transmission electron microscopy

Cells were treated as indicated, media removed and fixed in 0.1 M sodium cacodylate buffer (pH 7.2) containing 4% paraformaldehyde and 2.5% glutaraldehyde for 30 min at room temperature on the plate, scraped into fixative and left for a further 30 min at room temperature. Samples were then spun at 500× *g* for 15 min and the pellet washed twice in cacodylate buffer. For post‐fixation, samples were incubated in 1% OsO_4_ with 1.5% sodium ferrocyanide in 0.1 M cacodylate buffer for 60 min. Samples were subsequently stained with 1% tannic acid in 0.1 M cacodylate buffer for 1 h and washed in sodium acetate buffer (pH 5) overnight. Samples were then stained with 1% uranyl acetate in acetate buffer for 1 h and dehydrated in alcohol solution series from 50 to 100% with 10‐min incubation in each alcohol. Samples were changed to 100% propylene oxide with two times 10‐min incubations. Samples were changed to 50% propylene oxide, 50% Durcupan resin (Sigma; mix: A—5 g, B—5 g, C—6 drops, D—6 drops—invert to mix and avoid bubbles) and left overnight in rotator. The propylene oxide was then allowed to evaporate, and samples changed into 100% Durcupan resin in specimen embedding moulds, polymerized at 60°C overnight and sections cut on ultramicrotome at 70–100 nm thickness (Leica Ultracut UCT). Sections were stained with 3% aqueous uranyl acetate followed by Reynolds lead citrate. Grids were then imaged on JEOL 1200EX TEM using SIS camera and processed using FIJI (ImageJ).

### Lipid droplet induction and imaging

Cells were set up on glass coverslips and incubated with either complete medium plus 2% BSA or complete media plus 2% BSA/500 μM oleic acid for 16 h. Cells were then fixed in 4% PFA/PBS for 10 min and permeabilized using the saponin method detailed above. Coverslips were incubated with anti‐HA primary antibody and donkey anti‐rat Alexa 657 secondary antibody. Lipid droplets were stained using 5 μM BODIPY 493/503 (Thermo Fisher Scientific) to stain neutral lipids. Samples were mounted, imaged and analysed as detailed above.

### Protein expression and purification

GST‐tagged mammalian ATG8 fusion proteins were cloned into pGEX‐4T‐1 (GE Healthcare) and expressed in *Escherichia coli* BL21 (DE3) cells in LB medium as previously described 5. Expression was induced by addition of 0.5 mM IPTG, and cells were incubated at 16°C overnight. Harvested cells were lysed using sonication in a lysis buffer (20 mM Tris–HCl pH 7.5, 10 mM EDTA, 5 mM EGTA, 150 mM NaCl), and the supernatant was subsequently applied to Glutathione Sepharose 4B beads (GE Healthcare). After several washes, fusion protein‐bound beads were used directly in GST pull‐down assays.

### Proteinase K protection assay

Proteinase K assay was performed as previously detailed 60. Briefly, U2OS ATG2A/B DKO or DKO plus ATG2A‐WT, ATG2A‐mLIR or ATG2A‐mYFS cells were grown in complete media or starved (EBSS) in the presence of bafilomycin A1 (200 nM) for 4 h, scraped in PBS and centrifuged at 500× *g*. The cells were then resuspended in PBS/6.5 μg/ml digitonin incubated for 5 min at room temperature and then for a further 30 min on ice. Samples were subsequently centrifuged at 13,000× *g* and the supernatant removed. The membrane fractions were then resuspended in 50 mM Tris, pH 7.5, 0.18 M sucrose. Resuspended membrane pellets were then incubated with either buffer only, buffer + 100 ng/ml proteinase K (PK) or PK + 0.1% Triton X‐100 (PK+TX) for 10 min at 30°C. The reaction was stopped by addition of 3× Laemmli sample buffer and boiled at 95°C.

### Cloning and plasmid generation

pDONOR‐ATG2A and pDONOR‐ATG2B were kind gifts from C. Behrends. These were used in conjunction with Gateway cloning system (Invitrogen) pDEST‐CMV‐Myc and pMSCV‐Flag‐HA‐IRES‐Puro (iTAP) to generate plasmids expressing either ATG2A or ATG2B. Site‐directed mutagenesis was carried out to mutate the wild‐type gene for the required amino acid substitutions. For a full list of plasmids used in this study, see Table 2.

**Table 2:** Plasmids used in this study

Plasmid/epitope tag	Gene/mutation	Reference
pDONOR223‐hATG2A	Human ATG2A	This study
pDONOR223‐hATG2B	Human ATG2B	This study
(iTAP) pMSCV‐Flag‐HA‐ATG2A	ATG2A‐WT	This study
(iTAP) pMSCV‐Flag‐HA‐ATG2A‐mLIR	ATG2A aa1,362–1,365 FCIL/AAAA	This study
(iTAP) pMSCV‐Flag‐HA‐ATG2A‐mYFS	ATG2A aa1,395–1,397 YFS/AAA	This study
pDEST‐CMV‐Myc‐ATG2A‐WT	ATG2A‐WT	This study
pDEST‐CMV‐Myc‐ATG2A‐mLIR#1	ATG2A aa536–539 YTEI/AAAA	This study
pDEST‐CMV‐Myc‐ATG2A‐mLIR#2	ATG2A aa926–929 FSTL/AAAA	This study
pDEST‐CMV‐Myc‐ATG2A‐mLIR#3	ATG2A aa981–984 YFCL/AAAA	This study
pDEST‐CMV‐Myc‐ATG2A‐mLIR#4	ATG2A aa1,092–1,095 FLDV/AAAA	This study
pDEST‐CMV‐Myc‐ATG2A‐mLIR #5	ATG2A aa1,362–1,365 FCIL/AAAA	This study
pDEST‐CMV‐Myc‐ATG2B‐WT	ATG2B‐WT	This study
pDEST‐CMV‐Myc‐ATG2B‐mLIR#1	ATG2B aa845–848 WPRI/AAAA	This study
pDEST‐CMV‐Myc‐ATG2B‐mLIR#3	ATG2B aa979–982 FENI/AAAA	This study
pDEST‐CMV‐Myc‐ATG2B‐mLIR#4	ATG2B aa1,026–1,029 FSTV/AAAA	This study
pDEST‐CMV‐Myc‐ATG2B‐mLIR#5	ATG2B aa1,306–1,309 YVRV/AAAA	This study
pDEST‐CMV‐Myc‐ATG2B‐mLIR#6	ATG2B aa1,491–1,494 FCIL/AAAA	This study
pGEX‐4T1 alone	GST only	5
pGEX‐4T1‐LC3A‐ΔG	MAP1LC3A deletion of c‐term Gly	5
pGEX‐4T1‐LC3B‐ΔG	MAP1LC3B deletion of c‐term Gly	5
pGEX‐4T1‐LC3C‐ΔG	MAP1LC3C deletion of c‐term Gly	5
pGEX‐4T1‐GABARAP‐ΔG	GABARAP deletion of c‐term Gly	5
pGEX‐4T1‐GABARAP‐L1‐ΔG	GABARAP‐L1 deletion of c‐term Gly	5
pGEX‐4T1‐GABARAP L2‐ΔG	GABARAP‐L2 deletion of c‐term Gly	5
pEGFP‐C1 empty	GFP only	5
pEGFP‐C1‐LC3A	GFP‐tagged MAP1LC3A	5
pEGFP‐C1‐LC3B	GFP‐tagged MAP1LC3B	5
pEGFP‐C1‐LC3C	GFP‐tagged MAP1LC3C	5
pEGFP‐C1‐GABARAP	GFP‐tagged GABARAP	5
pEGFP‐C1‐GABARAP‐L1	GFP‐tagged GABARAP‐L1	5
pEGFP‐C1‐GABARAP‐L2	GFP‐tagged GABARAP‐L2 (GATE‐16)	5
pmCherry‐C1‐GABARAP	mCherry‐tagged GABARAP	This study
pDEST‐mCherry‐WIPI4	mCherry‐tagged WIPI4 (WDR45)	This study
pDEST‐EGFP‐WIPI4	GFP‐tagged WIPI4 (WDR45)	This study
pMRXIP‐GFP‐Stx17 WT	GFP‐tagged Syntaxin 17	51

## Author contributions

MB, LVDB, NG and ND performed experiments; BAM and LR characterized ATG2 KO cells; TJM designed and synthesized CRISPR/Cas9 guides; and ARP assisted in confocal imaging and prepared samples for TEM. DGM designed, performed and analysed experiments and wrote the manuscript.

## Conflict of interest

The authors declare that they have no conflict of interest.

## Supporting information



Expanded View Figures PDF

Movie EV1

Movie EV2

Source Data for Expanded View

Review Process File

Source Data for Figure 2
