# Supplementary material for: A conserved ATG2‐GABARAP family interaction is critical for phagophore formation
Source: EMBO Rep. 2020 Feb 3;21(3):e48412. doi: 10.15252/embr.201948412 (PMC7054675; doi:10.15252/embr.201948412)
Supplement: Supplementary file 1 — Expanded View Figures PDF [file EMBR-21-e48412-s001.pdf]

Expanded View Figures

Figure EV1. Generation of GFP-tagged endogenous ATG2A.

A, B Strategy for insertion of GFP-tag upstream of human ATG2A exon 1. Graphic shows position of guides and locus before and after (B) GFP-tag plus linker insertion.

C Western blot of total cell lysates from parental wild type (WT) and GFP-ATG2A CRISPR/Cas9 knock-in clones 1 and 2 using anti-ATG2A and anti-ATG2B antibodies.

D GFP alone or GFP-tagged mammalian ATG8 proteins (LC3A, LC3B, LC3C, GABARAP, GABARAP-L1 and GABARAP-L2) were overexpressed in HEK293T cells, lysed and the GFP-tag immunoprecipitated using GFP-TRAP beads. Samples were then run on 4–12% Bis–Tris gel and transferred to PVDF membrane and blotted for the presence of ATG2A, ATG2B, p62/SQSTM1, WIPI4 and anti-GFP. Blots are representative of n = 3 independent experiments.

Source data are available online for this figure.

 © 2020 The Authors

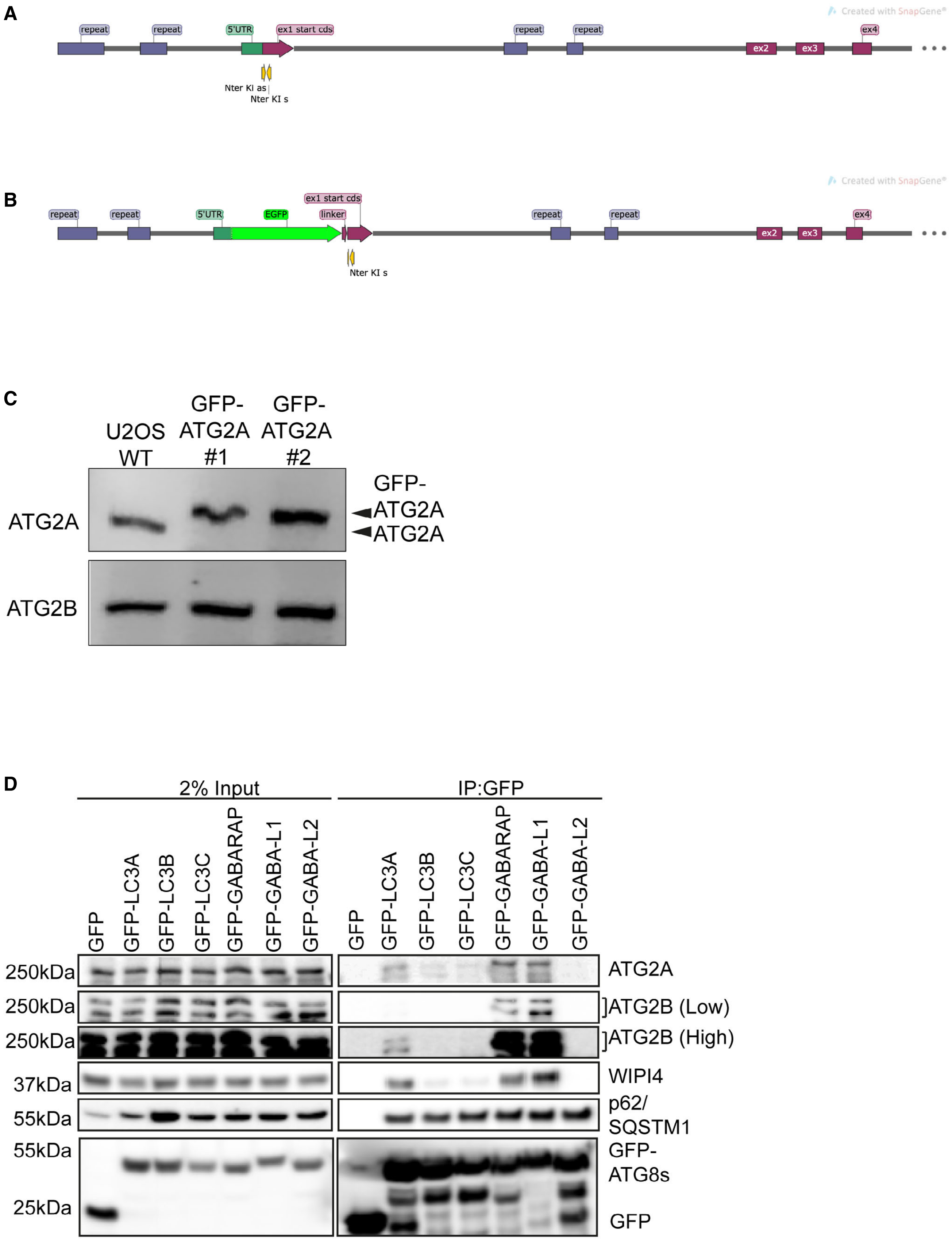
A
repeat
repeat
5'UTR
ex1 start cds
repeat
repeat
ex2
ex3
ex4
Nter KI as
Nter KI s
Created with SnapGene®
B
repeat
repeat
5'UTR
EGFP
linker
ex1 start cds
repeat
repeat
ex2
ex3
ex4
Nter KI s
Created with SnapGene®
C
U2OS WT
GFP-ATG2A #1
GFP-ATG2A #2
ATG2A
ATG2B
GFP-ATG2A
ATG2A
D
2% Input
IP:GFP
GFP
GFP-LC3A
GFP-LC3B
GFP-LC3C
GFP-GABARAP
GFP-GABA-L1
GFP-GABA-L2
250kDa
250kDa
250kDa
37kDa
55kDa
55kDa
25kDa
ATG2A
]ATG2B (Low)
]ATG2B (High)
WIPI4
p62/SQSTM1
GFP-ATG8s
GFP

Figure EV1.

Figure EV2. ATG2A and ATG2B contain a highly conserved LC3 interaction region (LIR). Related to Fig 2.

A Domain structure of ATG2A (green) and ATG2B (grey) proteins. Both ATG2s contain an N-terminal VPS13/chorein domain, ATG2 C-terminal autophagy domain (CAD motif) and ATG2 C-terminal domain. Position and sequence of putative ATG2 LC3 interaction regions (LIRs) as identified by iLIR and manual annotation. ATG2A has 5 potential LIRs, and ATG2B has 6 potential LIRs. See Table 1 for details.

B–E Myc-tagged ATG2A wild type (WT) and putative LIR mutants, where the potential core motif was mutated to alanine, were used in a pull-down assay with GST-tagged mammalian ATG8 proteins. Shown are ATG2-mLIR#1 (B), ATG2-mLIR#2 (C), ATG2-mLIR#3 (D) and ATG2-mLIR#4 (E). ATG2-mLIR#5 is shown on (H). ATG2A-WT or ATG2A-mLIRs were overexpressed in HEK293T cells, and lysates were incubated with purified GST alone or GST-tagged LC3A, LC3B, LC3C, GABARAP, GABARAP-L1 or GABARAP-L2. Samples were spun, washed and blotted for the presence/absence of Myc-tagged ATG2A using anti-Myc antibody. Anti-p62/SQSTM1 was used as an internal control for the GST pull-down samples. GST proteins were visualized by Ponceau S staining of membranes.

F, G As in (B) but using Myc-tagged ATG2B-WT or ATG2B-mLIR proteins. Shown are ATG2B mLIR#1 (F) and ATG2B mLIR#4 (G). ATG2B-mLIR#2 was present on an alpha helix, whereas ATG2B-mLIR#3 and ATG2B-mLIR#5 were not expressed. Anti-p62/SQSTM1 was used as an internal control for the GST pull-down samples. All blots are representative of at least $n = 3$ independent experiments.

H Myc-tagged ATG2A wild type (WT), ATG2A-mutant LIR #5 (mLIR; FCIL/AAAA) (upper blots) or Myc-tagged ATG2B-WT or ATG2B-mLIR (FCIL/AAAA; lower blots) were overexpressed in HEK293T cells, and lysates were incubated with purified GST alone or GST-tagged LC3A, LC3B, LC3C, GABARAP, GABARAP-L1 or GABARAP-L2. Samples were spun, washed and blotted for the presence/absence of Myc-tagged ATG2s using anti-Myc antibody. GST proteins were visualized by Ponceau S staining of membranes.

I U2OS WT and ATG2A/B double-knockout cell total lysates, analysed for the presence/absence of autophagy marker proteins including ATG2A, ATG2B, p62/SQSTM1, LC3A, LC3B, LC3C, GABARAP-L1, GABARAP-L2 and vinculin used as loading control.

J GFP alone, GFP-WIPI4 or GFP-WIPI4 with increasing concentrations of mCherry-GABARAP was expressed in HEK293T cells for 24 h, lysed and GFP-TRAP beads used to immunoprecipitate GFP alone or GFP-WIPI4. Samples were then probed with antibodies to detect endogenous ATG2A or ATG2B, anti-GFP and anti-GABARAP. Blots are representative of $n = 3$ independent experiments.

K Quantification of ATG2A (blue line, round symbols) and ATG2B (grey line and squares) co-precipitation with GFP-WIPI4 in the presence of increasing concentrations of mCherry-GABARAP from (J). Co-precipitation was normalized to GFP-WIPI4 alone. Line and error bars are mean $\pm$ SD of $n = 3$ independent experiments.

 © 2020 The Authors

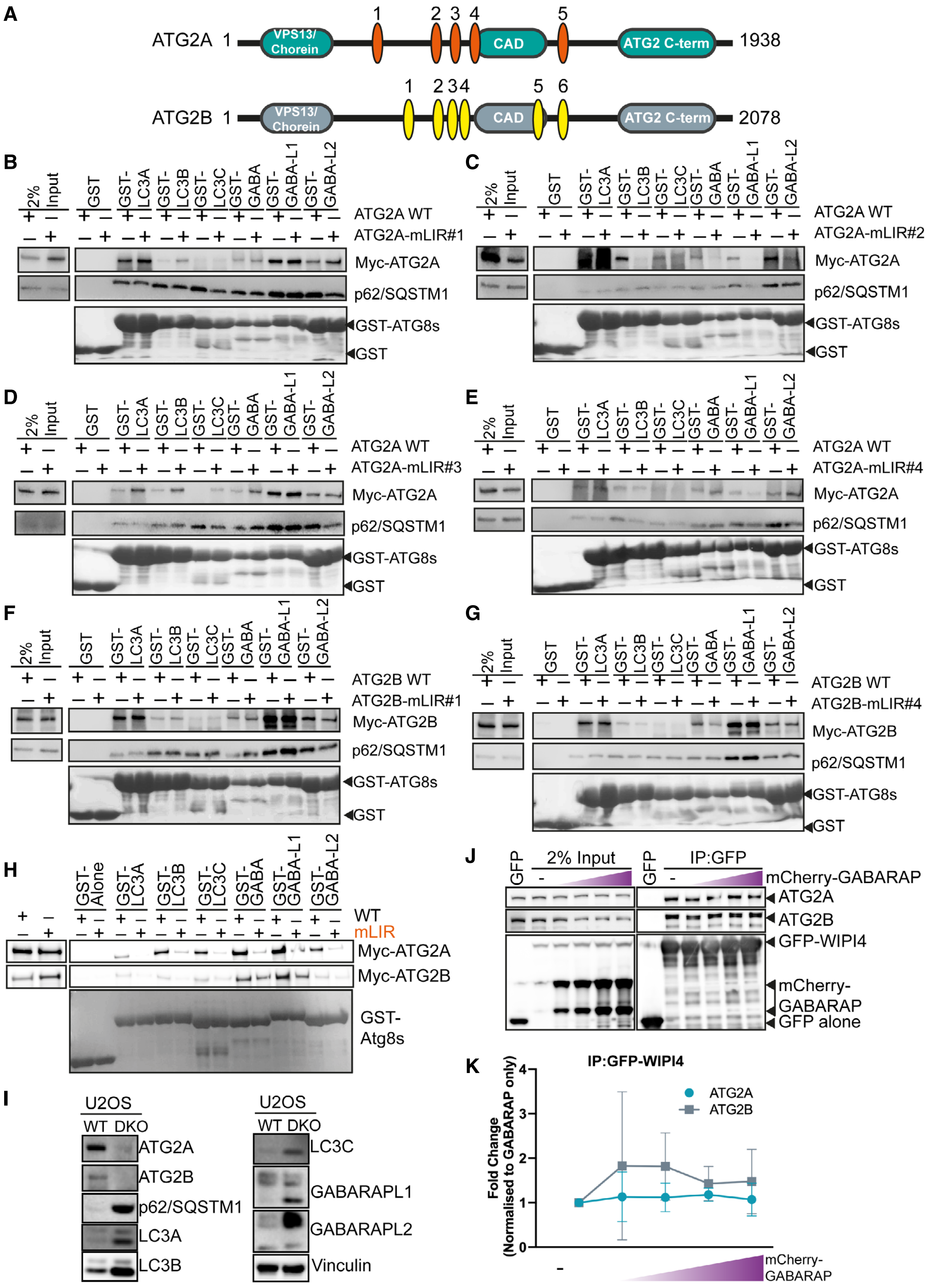
A
ATG2A 1
VPS13/ Chorein
CAD
ATG2 C-term
1938
ATG2B 1
2078
B
C
D
E
F
G
ATG2A WT
ATG2A-mLIR#1
ATG2A-mLIR#2
ATG2A-mLIR#3
ATG2A-mLIR#4
ATG2B WT
ATG2B-mLIR#1
ATG2B-mLIR#4
Myc-ATG2A
Myc-ATG2B
p62/SQSTM1
GST-ATG8s
GST
H
WT
mLIR
GST-Atg8s
I
U2OS
WT DKO
ATG2A
ATG2B
LC3A
LC3B
LC3C
GABARAPL1
GABARAPL2
Vinculin
J
2% Input
IP:GFP
mCherry-GABARAP
GFP-WIPI4
GFP alone
K
IP:GFP-WIPI4
Fold Change (Normalised to GABARAP only)

Figure EV2.

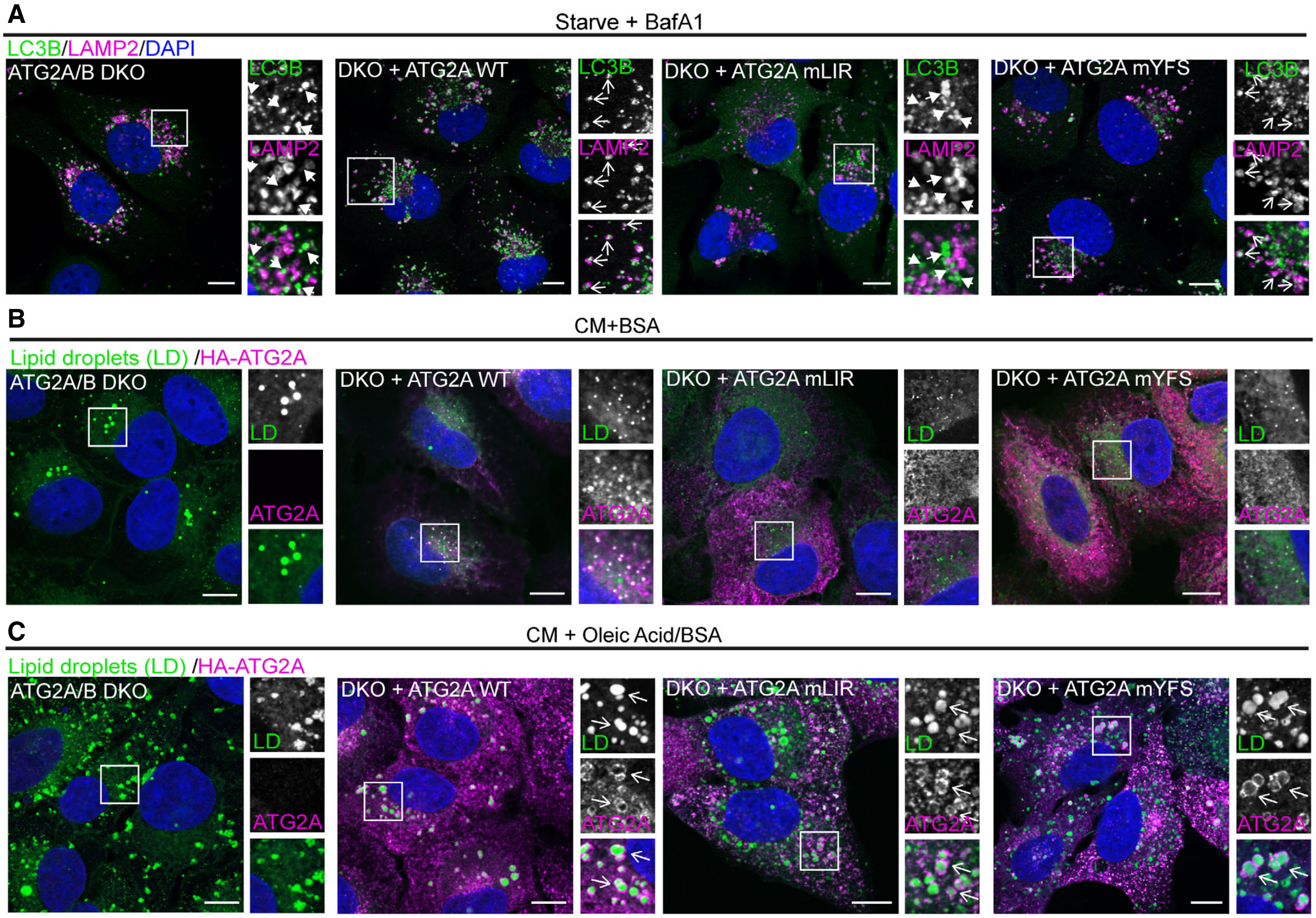

Figure EV3. ATG2A-WT and mutants effectively localize to lipid droplets. Related to Fig 3.

A U2OS ATG2A/B DKO cells reconstituted with vector only, HA-ATG2A-WT, HA-ATG2A-mLIR and HA-ATG2A-mYFS were stimulated starvation (EBSS) plus bafilomycin A1 (BafA1, 200 nM) for 4 h to stimulate autophagosome generation and prevent their degradation in the lysosome. Cells were fixed and immune-stained for LC3B (green) and LAMP2 (magenta) to visualize lysosomes. DAPI was included (blue) to mark the DNA/nucleus. Closed arrows (ATG2A/B DKO and DKO + ATG2A mLIR) highlight aggregate structures. Open arrows (DKO + ATG2A-WT and ATG2A-mYFS) highlight LAMP2/LC3B-positive vesicles. Scale bar 10 μm.

B, C U2OS ATG2A/B DKO cells reconstituted with vector only, HA-ATG2A-WT, HA-ATG2A-mLIR and HA-ATG2A-mYFS were stimulated with either 2% BSA only (B) or 2% BSA plus 500 μM oleic acid (C) for 16 h prior to fixation in 4% PFA. Cells were permeabilized using saponin and stained with anti-HA (ATG2A; magenta) and 5 μM BODIPY 493/503 to visualize lipid droplets (green; LDs). DAPI was included (blue) to mark the DNA/nucleus. Open arrows highlight ATG2A-positive lipid droplets. All images are representative of at least $n = 3$ independent experiments. Scale bar 10 μm.

Figure EV4. ATG2A-mLIR causes accumulation of early autophagy markers. Related to Fig 5.

A, B U2OS ATG2A/B DKO cells reconstituted with vector only, HA-ATG2A-WT, HA-ATG2A-mLIR and HA-ATG2A-mYFS were stimulated starvation (EBSS) 2 h to stimulate autophagosome generation. Cells were then fixed and immune-stained for LC3B (green), p62/SQSTM1 (magenta) (A) or GABARAP-L1 (magenta) (B) and DAPI (blue; DNA/nucleus). Images were taken on a Zeiss 880 AiryScan super-resolution confocal microscope. All images are representative of at least $n = 3$ independent experiments. Scale bar 10 μm.

C Total cell lysates of ATG2A/B double-knockout cells alone or reconstituted with HA-tagged ATG2A-WT, ATG2A-mLIR (FCIL/AAAA) or ATG2A-mYFS (YFS/AAA) and left in complete media (CM) or starved for 2 h (EBSS). Cells were lysed and blotted for autophagy marker proteins p62/SQSTM1, LC3B, ATG9A, WIPI2, GABARAP and GABARAP-L1. Anti-HA for ATG2A expression and vinculin were used as a loading control.

 © 2020 The Authors

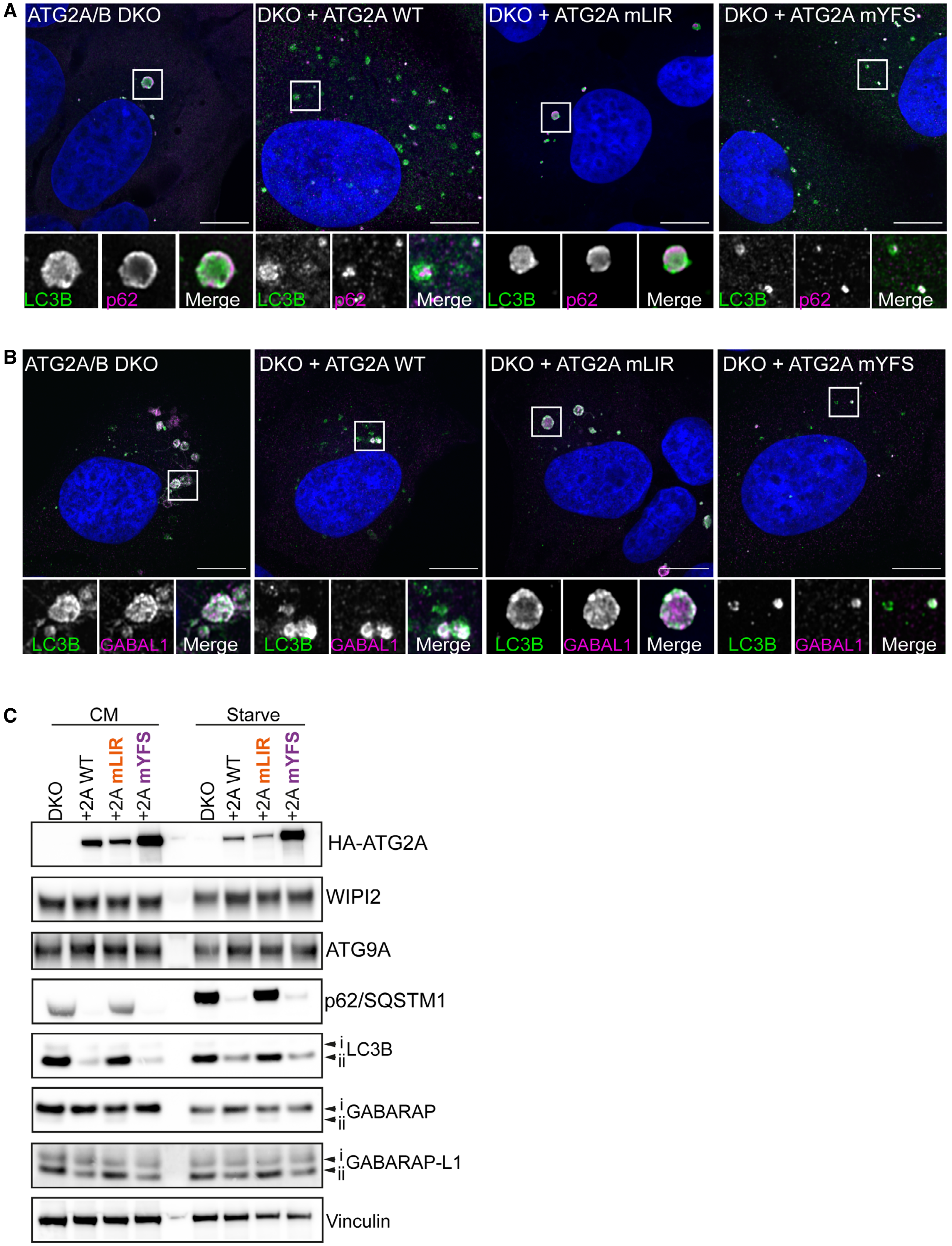
A
ATG2A/B DKO
DKO + ATG2A WT
DKO + ATG2A mLIR
DKO + ATG2A mYFS
LC3B
p62
Merge
B
ATG2A/B DKO
DKO + ATG2A WT
DKO + ATG2A mLIR
DKO + ATG2A mYFS
LC3B
GABAL1
Merge
C
CM
Starve
DKO
+2A WT
+2A mLIR
+2A mYFS
HA-ATG2A
WIPI2
ATG9A
p62/SQSTM1
i
ii
LC3B
GABARAP
GABARAP-L1
Vinculin

Figure EV4.
