# Supplementary material for: A conserved ATG2‐GABARAP family interaction is critical for phagophore formation
Source: EMBO Rep. 2020 Feb 3;21(3):e48412. doi: 10.15252/embr.201948412 (PMC7054675; doi:10.15252/embr.201948412)
Supplement: Supplementary file 2 — Movie EV1 [file EMBR-21-e48412-s002.zip › MovieEV1/MovieEV1_legend.docx]

**Movie EV1.** Airyscan confocal Z-stack of ATG2A/B double knockout cells stained with anti-LC3B (Green) and anti-GABARAP-L1 (Magenta). **Related to Figure EV4B.**
