# Supplementary material for: A conserved ATG2‐GABARAP family interaction is critical for phagophore formation
Source: EMBO Rep. 2020 Feb 3;21(3):e48412. doi: 10.15252/embr.201948412 (PMC7054675; doi:10.15252/embr.201948412)
Supplement: Supplementary file 4 — Source Data for Expanded View [file EMBR-21-e48412-s005.zip › embr201948412-sup-0005-SDataFigEV1D.pdf]

Figure EV1D

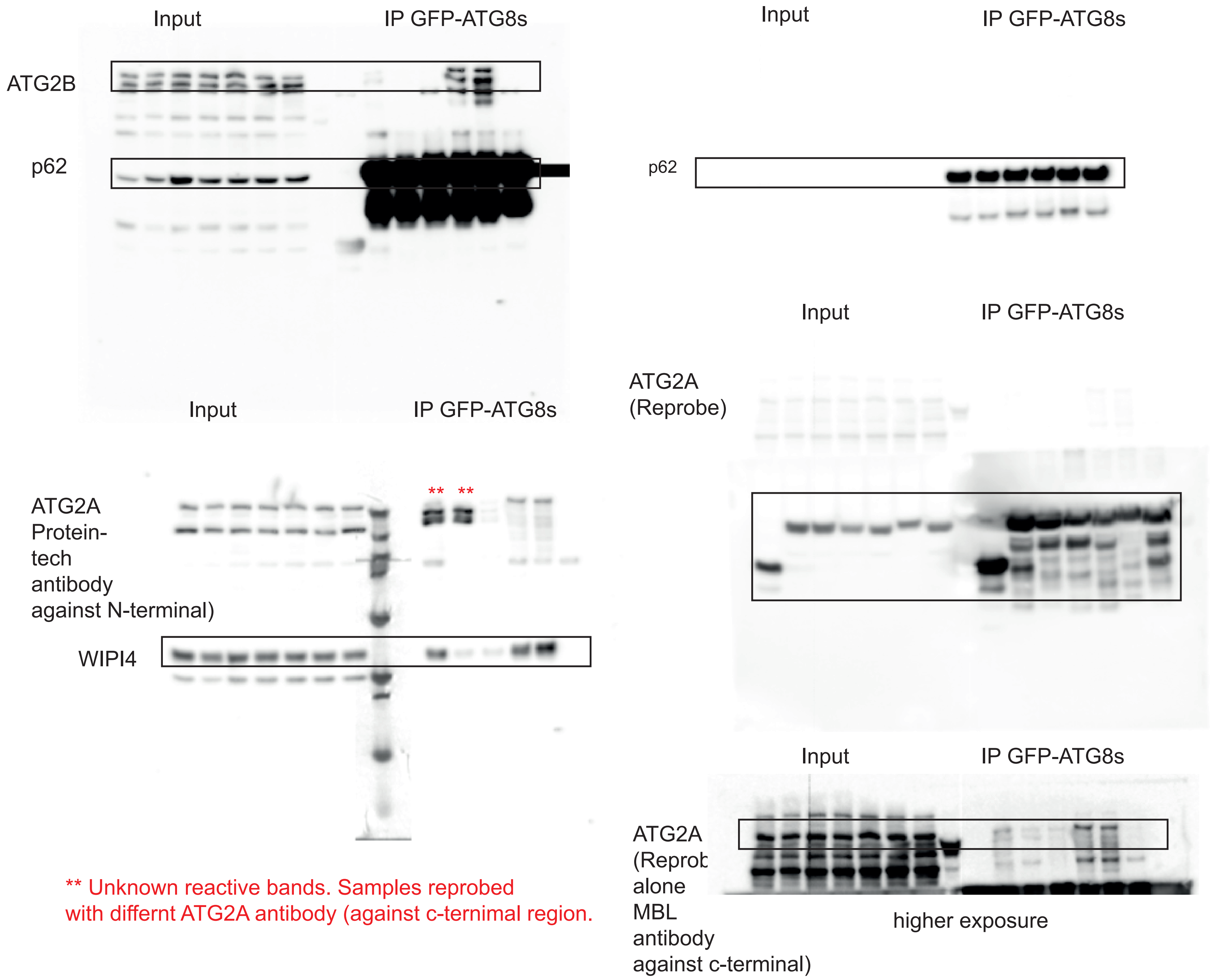
Input
IP GFP-ATG8s
ATG2B
p62
Input
IP GFP-ATG8s
p62
Input
IP GFP-ATG8s
ATG2A
Protein-
tech
antibody
against N-terminal)
**
**
WIPI4
Input
IP GFP-ATG8s
ATG2A
(Reprobe)
Input
IP GFP-ATG8s
ATG2A
(Reprob
alone
MBL
antibody
against c-terminal)
higher exposure
** Unknown reactive bands. Samples reprobed
with differnt ATG2A antibody (against c-ternimal region.
