# Supplementary figures and images for: A conserved ATG2‐GABARAP family interaction is critical for phagophore formation
Source: EMBO Rep. 2020 Feb 3;21(3):e48412. doi: 10.15252/embr.201948412 (PMC7054675; doi:10.15252/embr.201948412)

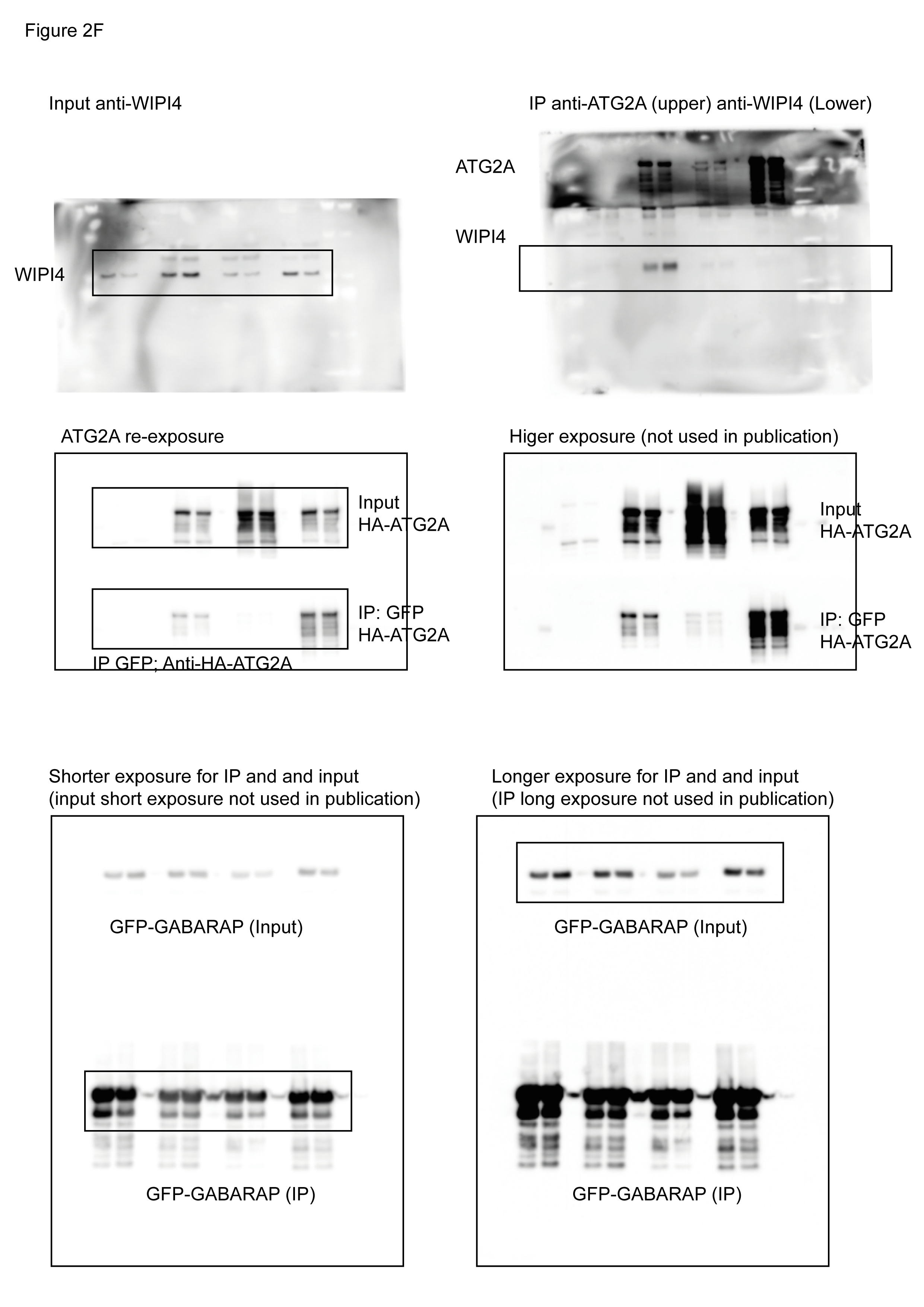

Supplement: Supplementary file 6 — Source Data for Figure 2 [file EMBR-21-e48412-s004.tif]
